# Fungal chitin-binding glycoprotein induces Dectin-2-mediated allergic airway inflammation synergistically with chitin

**DOI:** 10.1371/journal.ppat.1011878

**Published:** 2024-01-03

**Authors:** Yasunori Muraosa, Yutaro Hino, Shogo Takatsuka, Akira Watanabe, Emiko Sakaida, Shinobu Saijo, Yoshitsugu Miyazaki, Sho Yamasaki, Katsuhiko Kamei

**Affiliations:** 1 Division of Clinical Research, Medical Mycology Research Center, Chiba University, Chiba, Japan; 2 Department of Fungal Infection, National Institute of Infectious Diseases, Tokyo, Japan; 3 Department of Hematology, Chiba University Hospital, Chiba, Japan; 4 Division of Molecular Immunology, Medical Mycology Research Center, Chiba University, Chiba, Japan; 5 Division of Molecular Design, Medical Institute of Bioregulation, Kyushu University, Fukuoka, Japan; 6 Department of Molecular Immunology, Research Institute for Microbial Diseases, Osaka University, Osaka, Japan; 7 Laboratory of Molecular Immunology, Immunology Frontier Research Center, Osaka University, Osaka, Japan; 8 Division of Infection Control and Prevention, Medical Mycology Research Center, Chiba University, Chiba, Japan; 9 Department of Infectious Diseases, Japanese Red Cross Ishinomaki Hospital, Miyagi, Japan; University of Wisconsin-Madison, UNITED STATES

## Abstract

Although chitin in fungal cell walls is associated with allergic airway inflammation, the precise mechanism underlying this association has yet to be elucidated. Here, we investigated the involvement of fungal chitin-binding protein and chitin in allergic airway inflammation. Recombinant *Aspergillus fumigatus* LdpA (rLdpA) expressed in *Pichia pastoris* was shown to be an *O*-linked glycoprotein containing terminal α-mannose residues recognized by the host C-type lectin receptor, Dectin-2. Chitin particles were shown to induce acute neutrophilic airway inflammation mediated release of interleukin-1α (IL-1α) associated with cell death. Furthermore, rLdpA–Dectin-2 interaction was shown to promote phagocytosis of rLdpA–chitin complex and activation of mouse bone marrow-derived dendritic cells (BMDCs). Moreover, we showed that rLdpA potently induced T helper 2 (Th2)-driven allergic airway inflammation synergistically with chitin, and Dectin-2 deficiency attenuated the rLdpA–chitin complex-induced immune response *in vivo*. In addition, we showed that serum LdpA-specific immunoglobulin levels were elevated in patients with pulmonary aspergillosis.

## Author summary

Asthma is the most common chronic disease among children in many countries. Although its fundamental causes are not completely understood, the inhalation of allergens, including fungal spores is a known risk factor for the development of asthma. Recent studies have shown that sensitization to the filamentous fungus, *Aspergillus fumigatus*, is associated with exacerbation of allergic asthma and other pulmonary diseases. Although chitin in fungal cell walls is associated with allergic airway inflammation, the precise mechanism underlying this association has yet to be elucidated. In this study, we showed that *A*. *fumigatus* chitin-binding glycoprotein, LysM domain protein A (LdpA), synergistically induces Dectin-2-mediated Th2-driven airway inflammation with chitin. Therefore, this study provided new insights into fungal-induced allergic airway diseases.

## Introduction

Asthma is the most common chronic disease among children, and was reported to affect more than 262 million people worldwide in 2019 [1]. Although its fundamental causes are not completely understood, the inhalation of allergens, including fungal spores, pollen, and house dust mites, is a known risk factor for the development of asthma. Recent studies have shown that sensitization to the filamentous fungus, *Aspergillus fumigatus*, is associated with exacerbation of allergic asthma and other pulmonary diseases [2–5]. *A*. *fumigatus* is also known to cause allergic bronchopulmonary aspergillosis (ABPA) and allergic *Aspergillus* sinusitis (AAS) [6–9].

Previous studies have suggested that the fungal cell wall chitin, an *N*-acetyl-d-glucosamine (GlcNAc) polymer, is associated with allergic airway inflammation [10,11]. Chitin has been shown to exert size-dependent effects on innate immune responses [12]. Phagocytable-size chitin particles were shown to induce the production of proinflammatory cytokines, such as tumor necrosis factor (TNF)-α *in vitro*, whereas larger chitin particles did not induce such a response [13–18]. In previous *in vivo* studies, non-phagocytable-size chitin particles were shown to induce innate eosinophilic infiltration into the lungs of mice mediated by the release of epithelial cell-derived cytokines, such as thymic stromal lymphopoietin (TSLP), interleukin-25 (IL-25), and IL-33, with subsequent activation of innate lymphoid type 2 cells (ILC2s) [13,19–21]. In addition, chitin is known to act as an immune adjuvant that enhances the adaptive immune response [22–27].

The C-type lectin receptor (CLR), Dectin-2, is expressed by various myeloid cell populations, including macrophages and several dendritic cell subsets, where it interacts with α-1,2-mannose structures on fungi and glycans on house dust mite allergens [28–30] The recognition of house dust mite allergens by Dectin-2 was shown to induce T helper 2 (Th2) cell immunity [31,32], but the involvement of Dectin-2 in fungal-induced allergic immune responses has not been elucidated.

Previously, we reported that the *A*. *fumigatus* cell wall protein, LysM domain protein A (LdpA), which possesses multiple chitin-binding LysM domains, showed no functional characteristics that would affect fungal morphology or pathogenicity [33]. Hyphae, resting conidia, and swollen conidia showed *ldpA* mRNA expression, with higher levels of expression in hyphae [33]. In this study, we investigated the involvement of fungal chitin-binding protein and chitin in allergic airway inflammation using recombinant *A*. *fumigatus* LdpA expressed in *Pichia pastoris* (rLdpA). The results showed that rLdpA is an *O*-linked glycoprotein containing the terminal α-mannose residues recognized by the host CLR, Dectin-2. Phagocytable-size chitin particles were shown to induce acute neutrophilic airway inflammation mediated by cell death-associated IL-1α release. Furthermore, we found that the rLdpA–Dectin-2 interaction promotes phagocytosis of rLdpA–chitin complex and activation of mouse bone marrow-derived dendritic cells (BMDCs). Finally, we showed that rLdpA synergistically induces Dectin-2-mediated Th2-driven airway inflammation with chitin.

## Results

### Generation of recombinant LdpA in *Pichia pastoris*

**Fig 1:**
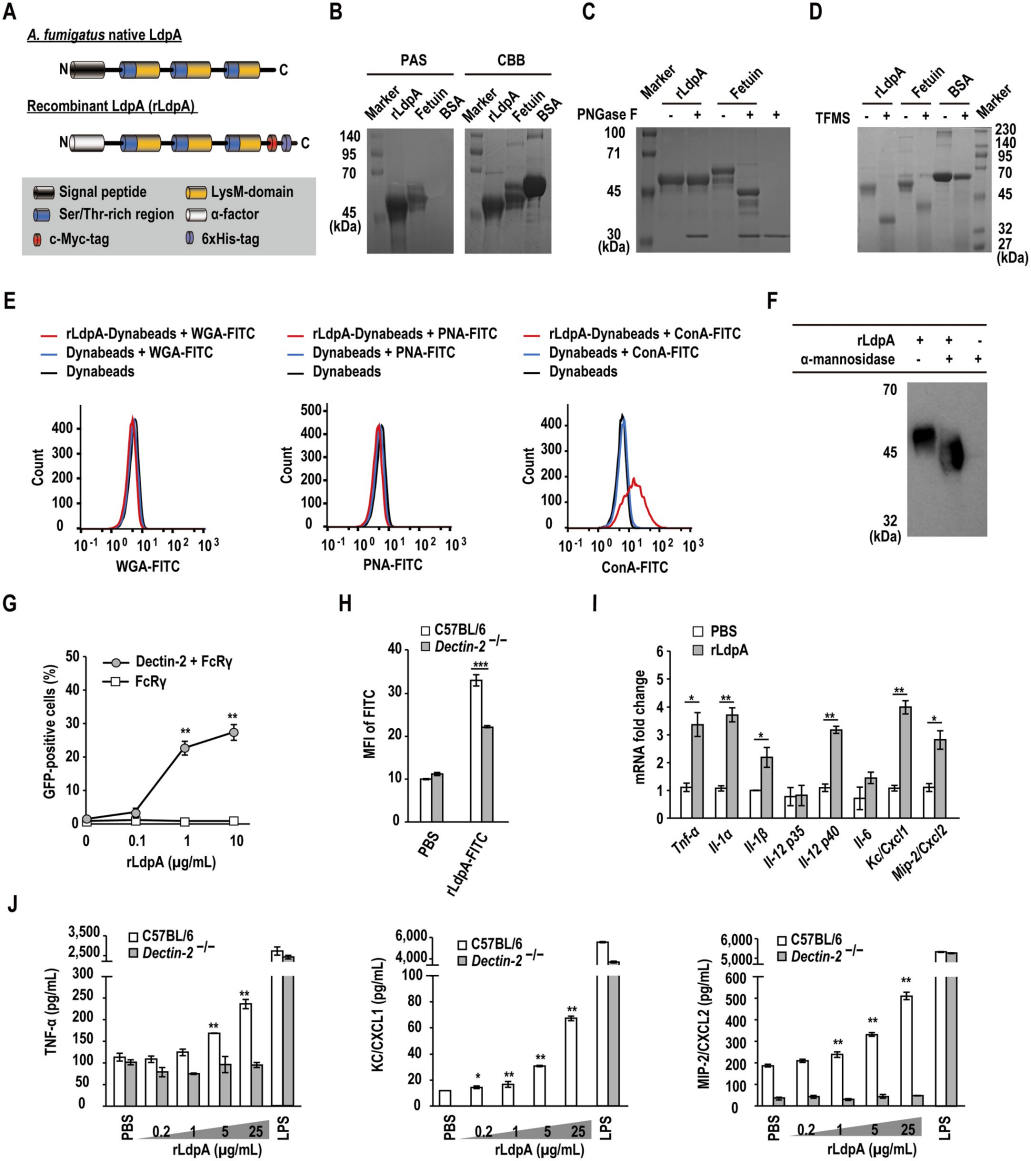
rLdpA, a glycoprotein with terminal α-mannose residues, induces Dectin-2-mediated proinflammatory cytokines and chemokines. (A) Domain organization of *A*. *fumigatus* native LdpA and recombinant LdpA (rLdpA) expressed in *Pichia pastoris*. (B) Glycoprotein staining of rLdpA. rLdpA protein was stained with periodic acid–Schiff (PAS) and Coomassie brilliant blue (CBB). Bovine fetuin and bovine serum albumin (BSA) were used as glycoprotein and non-glycoprotein controls, respectively. (C) Deglycosylation of *N*-linked glycan. After treatment of rLdpA with or without PNGase F, gel mobility shift assay on sodium dodecyl sulfate-polyacrylamide gel electrophoresis (SDS-PAGE) with CBB staining was performed. Bovine fetuin was used as a glycoprotein control. (D) Deglycosylation of *N*-linked and *O*-linked glycans. After treatment of rLdpA with or without trifluoromethanesulfonic acid (TFMS), gel mobility shift assay on SDS-PAGE with CBB staining was performed. Bovine fetuin and BSA were used as glycoprotein and non-glycoprotein controls, respectively. (E) Lectin-binding assays. rLdpA (20 μg) was reacted with 1 mg of Dynabeads to generate rLdpA–Dynabeads complex. After incubation of rLdpA–Dynabeads complex with fluorescein isothiocyanate (FITC)-labeled concanavalin A (ConA) (20 μg), FITC-labeled wheatgerm agglutinin (WGA) (20 μg), and FITC-labeled peanut agglutinin (PNA) (20 μg), lectins bound to rLdpA were measured by flow cytometry. (F) Deglycosylation of α-mannose residues. After treatment of rLdpA with or without α-mannosidase, which has exoglycosidase activity and removes terminal α1-2-, α1-3-, and α1-6-linked mannose residues from oligosaccharides, gel mobility shift on SDS-PAGE was determined by Western blotting analysis with anti-c-Myc antibody. (G) Dectin-2-NFAT-GFP reporter assay. NFAT-GFP reporter cells expressing FcRγ with or without Dectin-2 were incubated on plates coated with rLdpA (0.1, 1, and 10 μg/mL) for 24 h, and GFP-positive cells were measured by flow cytometry. (H) After bone marrow-derived dendritic cells (BMDCs) derived from C57BL/6 mice or *Dectin-2*^−/−^ mice were incubated with 25 μg/mL FITC-labeled rLdpA for 1 h, the mean fluorescence intensity (MFI) of cells was measured by flow cytometry. (I) Proinflammatory cytokine and chemokine mRNA expression. BMDCs derived from C57BL/6 mice were incubated with 25 μg/mL of rLdpA for 1.5 h, and then *Tnf-α*, *Il-1α*, *Il-1β*, *Il-6*, *Il-12 p35*, *Il-12 p40*, *Kc/Cxcl1*, and *Mip-2/Cxcl2* mRNA expression levels were measured by quantitative real-time PCR. The mRNA expression levels were normalized relative to *Gapdh* mRNA and are shown as fold change relative to the control phosphate-buffered saline (PBS) group. (J) Proinflammatory cytokine and chemokine protein levels. BMDCs derived from C57BL/6 and *Dectin-2*^−/−^ mice were incubated with 0.2–25 μg/mL rLdpA and 5 ng/mL lipopolysaccharide (LPS) for 24 h, TNF-α, KC/CXCL1, and MIP-2/CXCL2 protein levels in the culture supernatant were measured by enzyme-linked immunosorbent assay (ELISA). Data are shown as the mean ± standard deviation (SD). *P < 0.05, **P < 0.01, and ***P < 0.001 by unpaired two-tailed Student’s *t* test (H and I) or one-way ANOVA with Dunnett’s multiple comparison test (G and J).

C-terminal c-Myc- and 6×histidine (6×His)-tagged recombinant LdpA (rLdpA) protein, in which the *A*. *fumigatus* native signal peptide had been replaced with the alpha-factor secretion signal from *Saccharomyces cerevisiae*, was heterologously expressed in the methylotrophic yeast, *Pichia pastoris* (Fig 1A). After induction of rLdpA expression with methanol, the total protein level in the culture supernatant was increased in the *ldpA* transformant, GS115-LdpA (S1A Fig). After His-tag purification, a single protein band was confirmed by sodium dodecyl sulfate-polyacrylamide gel electrophoresis (SDS-PAGE) and Western blotting analysis (S1B and S1C Fig).

### rLdpA is an *O*-linked glycoprotein containing α-mannose residues

Prediction of *O*-linked glycosylation was performed using NetOGlyc 4.0 server (http://www.cbs.dtu.dk/services/NetOGlyc/) [34]. *A*. *fumigatus* LdpA was shown to contain putative *O*-glycosylation sites, which were rich in serine (Ser) and threonine (Thr) residues, at the N-terminal end of each LysM domain (Fig 1A). Ser and Thr residues accounted for 56.2%–66.6% of the amino acid content in the Ser/Thr-rich regions. Periodic acid–Schiff (PAS) staining, which stains glycoproteins, was performed to determine the glycosylation of rLdpA. Both rLdpA and bovine fetuin as a glycoprotein control showed positive staining with PAS, while bovine serum albumin (BSA) as a non-glycoprotein control showed no staining (Fig 1B). Glycosylation of rLdpA was also examined by gel mobility shift assay after treatment with PNGase F, which removes almost all *N*-linked oligosaccharides from glycoproteins. SDS-PAGE of PNGase-F-treated rLdpA showed no shift in mobility on the gel as an indicator of change in molecular mass (M_r_) (Fig 1C), while rLdpA showed a clear shift in mobility after treatment with trifluoromethanesulfonic acid (TFMS), which has been used extensively to chemically remove *N*- and *O*-linked oligosaccharides from glycoproteins (Fig 1D) [35,36]. The M_r_ of deglycosylated rLdpA treated with TFMS corresponded to its predicted M_r_ of 38 kDa. Next, the binding ability of rLdpA to lectins was evaluated. The results indicated that fluorescein isothiocyanate (FITC)-labeled concanavalin A (ConA), an α-mannose-binding lectin, bound to rLdpA, while FITC-labeled wheatgerm agglutinin (WGA) and peanut agglutinin (PNA) showed no binding (Fig 1E). Next, we examined the mannosylation of rLdpA by treatment with α-mannosidase, which has exoglycosidase activity and removes terminal α1-2-, α1-3-, and α1-6-linked mannose residues from oligosaccharides. After treatment with α-mannosidase, rLdpA showed a clear shift in its M_r_ (Fig 1F). Taken together these observations suggested that rLdpA is an *O*-linked glycoprotein with terminal α-mannose residues.

### rLdpA is a Dectin-2 ligand that induces proinflammatory cytokines and chemokines

As the CLR, Dectin-2, recognizes fungal α-mannan [28], we performed Dectin-2–NFAT–GFP reporter assay to investigate the interaction between rLdpA and Dectin-2 [37]. The results showed that rLdpA delivered dose-dependent activation signals *via* Dectin-2 (Fig 1G). Internalization of FITC-labeled rLdpA by BMDCs derived from *Dectin-2*^−/−^ mice was attenuated in comparison to BMDCs derived from C57BL/6 mice (Fig 1H). In addition, rLdpA was shown to induce proinflammatory cytokine (*Tnf-α*, *Il-1α*, *Il-1β*, *Il-6*, *Il-12 p40*) and chemokine (*Kc/Cxcl1 and Mip-2/Cxcl2*) mRNA expression (Fig 1I), and the TNF-α, KC/CXCL1, and MIP-2/CXCL2 protein levels in the culture supernatant of BMDCs incubated with rLdpA for 24 h were also increased in an rLdpA dose-dependent manner (Fig 1J). The findings outlined above suggested that rLdpA induces proinflammatory cytokine and chemokine expression *via* the CLR, Dectin-2.

### Phagocytosis of chitin particles by BMDCs induces IL-1α release after cell death

**Fig 2:**
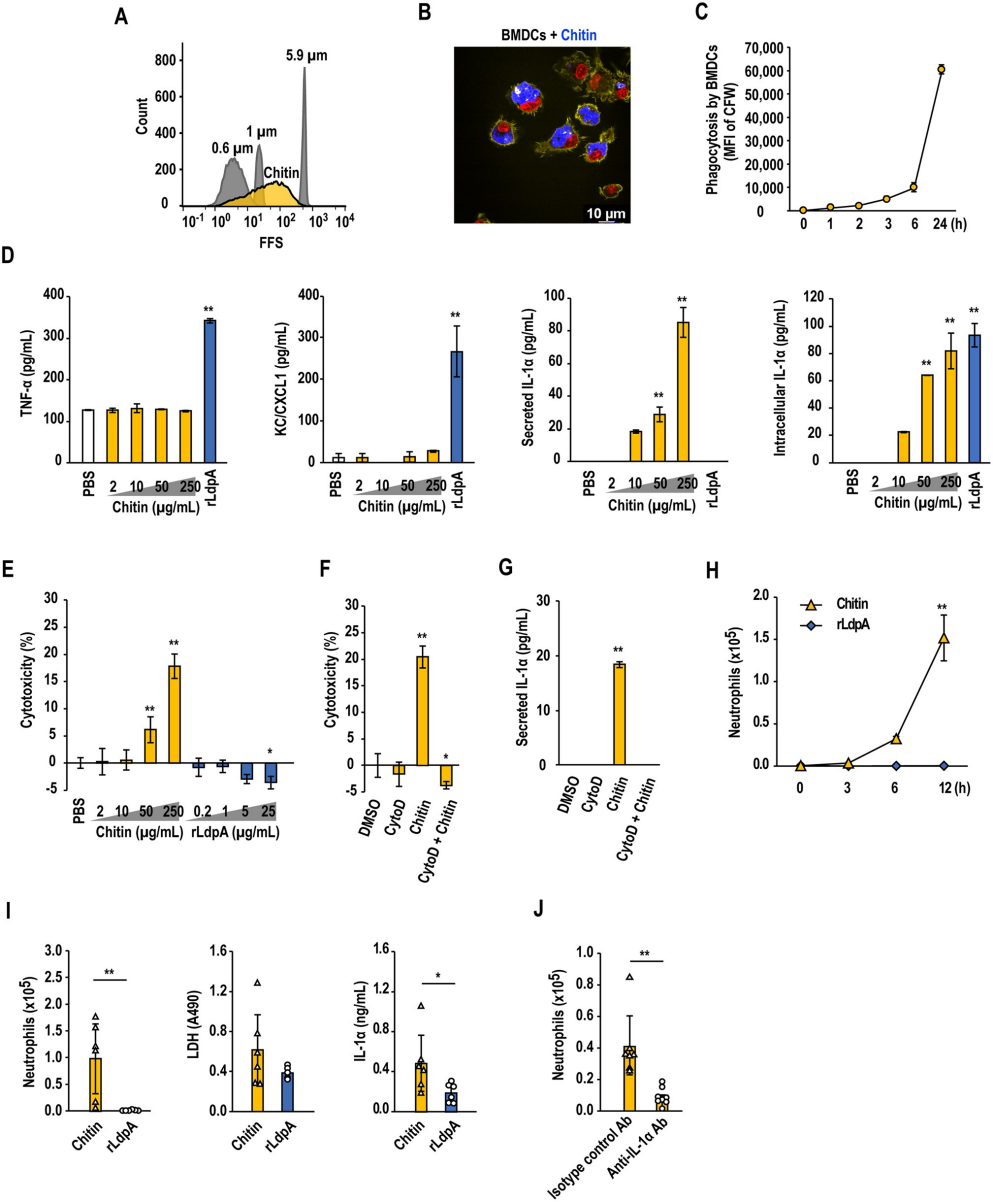
Phagocytosis of chitin particles induced IL-1α release after cell death and acute neutrophilic airway inflammation. (A) Flow cytometric analysis of chitin particle size. The yellow peak indicates chitin particles. Gray peaks indicate 0.6-, 1-, and 5.9-μm latex beads. (B, C) Phagocytosis assay of chitin particles. Bone marrow-derived dendritic cells (BMDCs) derived from C57BL/6 mice were incubated with 50 μg/mL of calcofluor white (CFW)-labeled chitin particles (blue). After washing with phosphate-buffered saline (PBS), cells were stained with Alexa Fluor 555 Phalloidin (yellow) and nuclei were stained with NucRed Live 647 ReadyProbes Reagent (red), and observed by confocal laser scanning microscopy (B). The mean fluorescence intensity (MFI) of cells that had phagocytosed chitin particles was measured by flow cytometry (C). (D, E) BMDCs were incubated with chitin particles (2–250 μg/mL), rLdpA (25 μg/mL), or PBS (vehicle control), and TNF-α, KC/CXCL1, and IL-1α levels in the culture supernatant and IL-1α in the cell lysate were measured by enzyme-linked immunosorbent assay (ELISA). Cytotoxicity was assessed by measuring the release of lactate dehydrogenase (LDH) (E). (F, G) BMDCs were incubated with 1 μM cytochalasin D for 30 min, followed by the addition of chitin particles (250 μg/mL). After 24-h incubation, cytotoxicity was assessed by measuring the release of LDH (F), and IL-1α protein levels in the culture supernatant were measured by ELISA (G). (H) C57BL/6 mice were administered chitin particles (100 μg), rLdpA (10 μg), or PBS (vehicle control) in a single intranasal dose. Bronchoalveolar lavage (BAL) fluid samples were obtained after 3, 6, and 12 h, and the numbers of neutrophils were measured by flow cytometry. (I) Chitin particles (100 μg) and rLdpA (10 μg) were administered to C57BL/6 mice in a single intranasal dose. The number of neutrophils, IL-1α level, and LDH level in the BAL fluid were measured after 24 h. (J) Anti-IL-1α neutralizing antibody or isotype control antibody was injected into the peritoneal cavity of C57BL/6 mice at a dose of 80 μg/mouse 1 h before intranasal chitin particle administration. The number of neutrophils in the BAL fluid was determined after 24 h. Data are shown as the mean ± standard deviation (SD). (H, *n* = 3 mice/group; I, *n* = 6 mice/group; J, *n* = 7–8 mice/group). Each symbol represents an individual sample (I and J). *P < 0.05 and **P < 0.01 by one-way ANOVA with Dunnett’s multiple comparison test (D–H) or unpaired two-tailed Student’s *t* test (I and J).

Here, we examined the phagocytosis of chitin particles approximately 0.6–5.9 μm in size (Fig 2A). Calcofluor white (CFW)-labeled chitin particles were phagocytosed by BMDCs (Fig 2B and 2C), but did not induce TNF-α or KC/CXCL1 production (Fig 2D). Interestingly, phagocytosis of chitin particles induced the production of IL-1α, a typical alarmin (Fig 2D). IL-1α is constitutively expressed at a steady state in many cell types in healthy tissues, and its expression can be increased in response to proinflammatory stimuli [38]. Necrotic cell death due to damage, stress, or infection induces the release of IL-1α into the surrounding milieu, which triggers the IL-1α-driven inflammatory loop [38]. In the present study, phagocytosis of chitin particles by BMDCs induced IL-1α release and cell death (Fig 2E and 2D), and inhibition of phagocytosis by cytochalasin D abolished both chitin particle-induced cell death (Fig 2F) and IL-1α release (Fig 2G). In contrast to chitin particles, BMDCs readily phagocytosed polystyrene- and cobalt-based beads (Dynabeads; Thermo Fisher Scientific, Waltham, MA, USA) (S2A Fig), which did not induce cell death or IL-1α release (S2B and S2C Fig). Taken together, these observations suggested that phagocytosis of chitin particles triggers innate inflammation by inducing IL-1α production and cell death-mediated IL-1α release.

### Chitin particles induce IL-1α-mediated acute neutrophilic airway inflammation *in vivo*

We next investigated the effects of chitin particles and rLdpA on airway inflammation *in vivo*. Chitin particles induced acute neutrophil infiltration into the airways of mice 6 hours after intranasal administration of a single dose, while rLdpA did not induce acute neutrophilic airway inflammation (Fig 2H). Lactate dehydrogenase (LDH) and IL-1α levels in the BAL fluid were elevated in the mice administered chitin particles (Fig 2I). The induction of acute neutrophil infiltration by chitin particles was inhibited by intraperitoneal premedication with anti-IL-1α neutralizing antibody (Fig 2J). These results suggested that IL-1α is a critical cytokine in chitin particle-induced acute neutrophilic airway inflammation.

### rLdpA–Dectin-2 interaction promotes phagocytosis of rLdpA–chitin complex

**Fig 3:**
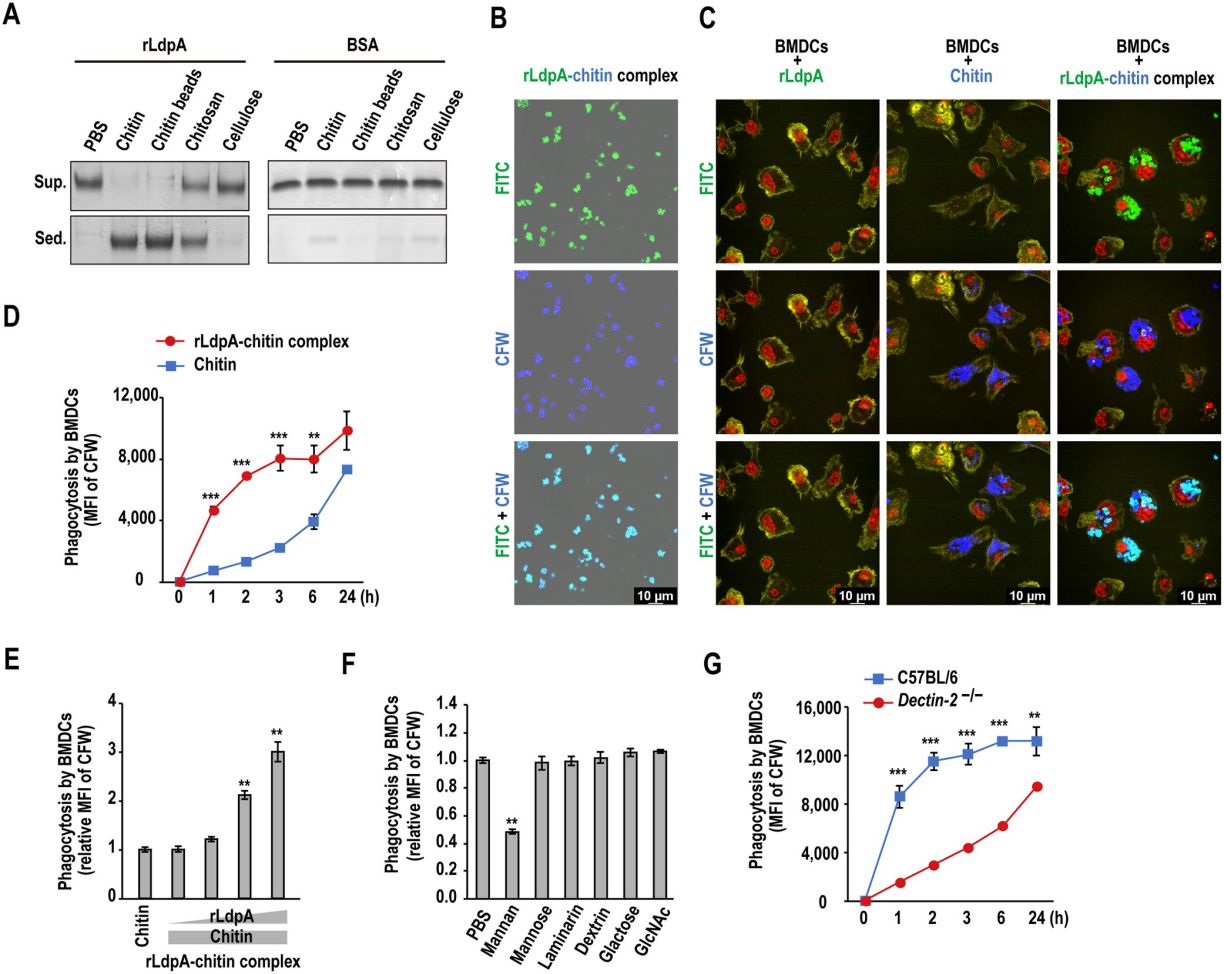
rLdpA–Dectin-2 interaction promotes phagocytosis of the rLdpA–chitin complex. (A) Affinity of recombinant LdpA (rLdpA) for insoluble polysaccharides determined by sedimentation assay. rLdpA (10 μg) and BSA (10 μg) were incubated with insoluble polysaccharides, chitin (1 mg), chitin beads (1 mg), chitosan (1 mg) (≥ 80.0% deacetylation), and cellulose (1 mg) in 100 μL of phosphate-buffered saline (PBS). rLdpA in the supernatant (sup.) and sediment (sed.) was detected by sodium dodecyl sulfate-polyacrylamide gel electrophoresis (SDS-PAGE). (B) Fluorescein isothiocyanate (FITC)-labeled rLdpA (20 μg) was reacted with 1 mg of calcofluor white (CFW)-labeled chitin. The resulting rLdpA–chitin complex was observed by confocal laser scanning microscopy. (C) Phagocytosis assay of rLdpA–chitin complex. Mouse bone marrow-derived dendritic cells (BMDCs) derived from C57BL/6 mice were incubated with FITC-labeled rLdpA (1 μg/mL), CFW-labeled chitin (50 μg/mL), and FITC- and CFW-labeled rLdpA–chitin complex (rLdpA, 1 μg/mL; chitin, 50 μg/mL) for 4 h. After washing with PBS, cells were stained with Alexa Fluor 555 Phalloidin (yellow), nuclei were stained with NucRed Live 647 ReadyProbes Reagent (red), and observed by confocal laser scanning microscopy. (D) BMDCs derived from C57BL/6 mice were incubated with CFW-labeled rLdpA–chitin complex (rLdpA, 1 μg/mL; chitin, 50 μg/mL) and CFW-labeled chitin (50 μg/mL) for 1–24 h. After washing with PBS, the mean fluorescence intensity (MFI) of rLdpA–chitin complex-phagocytosed cells was measured by flow cytometry. (E) Effects of LdpA on the phagocytosis of rLdpA–chitin complex. CFW-labeled chitin (1 mg) was reacted with different amounts of rLdpA (0.16–20 μg) for 30 min. The resulting rLdpA–chitin complexes were incubated with BMDCs derived from C57BL/6 mice for 4 h and then washed with PBS. The mean fluorescence intensity (MFI) of rLdpA–chitin complex-phagocytosed cells was measured by flow cytometry. (F) Inhibition assay of rLdpA-chitin complex phagocytosis. BMDCs derived from C57BL/6 mice were incubated with 1 mg/mL mannan, 1 mg/mL mannose, 1 mg/mL laminarin, 1 mg/mL dextran, 1 mg/mL galactose, 1 mg/mL *N*-acetyl-glucosamine (GlcNAc), and PBS for 30 min, followed by addition of CFW-labeled rLdpA–chitin complex (rLdpA, 1 μg/mL, chitin, 50 μg/mL). After 4 h of incubation, the MFI of rLdpA–chitin complex-phagocytosed cells was measured by flow cytometry. (G) BMDCs derived from C57BL/6 mice and *Dectin-2*^−/−^ mice were incubated with CFW-labeled rLdpA–chitin complex (rLdpA, 1 μg/mL; chitin, 50 μg/mL) for 1–24 h. After washing with PBS, the MFI of rLdpA–chitin complex-phagocytosed cells was measured by flow cytometry. Data are shown as the mean ± standard deviation (SD). *P < 0.05, **P < 0.01, and ***P < 0.001 by unpaired two-tailed Student’s *t* test (D and G) or one-way ANOVA with Dunnett’s multiple comparison test (E and F).

LdpA contains three chitin-binding LysM domains and is localized within the cell wall and extracellular matrix [33], suggesting that LdpA is a chitin-associated protein. In sedimentation assay using various insoluble polysaccharides, rLdpA was shown to bind to chitin particles and chitin beads, and approximately 50% of the rLdpA bound to chitosan, an 80% deacetylated product of chitin (Fig 3A). These observations suggested that LdpA is a chitin-binding protein. We quantified the chitin-binding capacity of rLdpA, and the results showed that approximately 10–20 μg of rLdpA bound to 1 mg of chitin (S3 Fig). Next, we investigated the effects of rLdpA–chitin complex on BMDCs. First, fluorescence-labeled rLdpA–chitin complex was generated by reacting FITC-labeled rLdpA and CFW-labeled chitin particles (Fig 3B). The results of phagocytosis assay showed that BMDCs readily phagocytosed rLdpA–chitin complex compared with chitin, indicating that rLdpA enhanced the phagocytosis of rLdpA–chitin complex (Fig 3C and 3D). To further examine the promotion of phagocytosis by rLdpA, different concentrations of rLdpA (0.0012–20 μg) were reacted with a specific amount of CFW-labeled chitin. The phagocytosis of rLdpA–chitin complex by BMDCs was shown to be increased in an rLdpA concentration-dependent manner (Fig 3E). We next examined whether the LdpA–Dectin-2 interaction contributed to the phagocytosis of rLdpA–chitin complex. Briefly, BMDCs were incubated with various carbohydrate ligands of CLRs prior to the addition of rLdpA–chitin complex. The phagocytosis of rLdpA–chitin complex by BMDCs was inhibited by the Dectin-2 ligand, mannan from *Saccharomyces cerevisiae* (Fig 3F). Furthermore, the phagocytosis of rLdpA–chitin complex was attenuated in BMDCs from *Dectin-2*^−/−^ mice (Fig 3G). These results indicated that the rLdpA–Dectin-2 interaction promotes phagocytosis of rLdpA–chitin complex.

### rLdpA–chitin complex activates BMDCs in a Dectin-2-dependent manner

**Fig 4:**
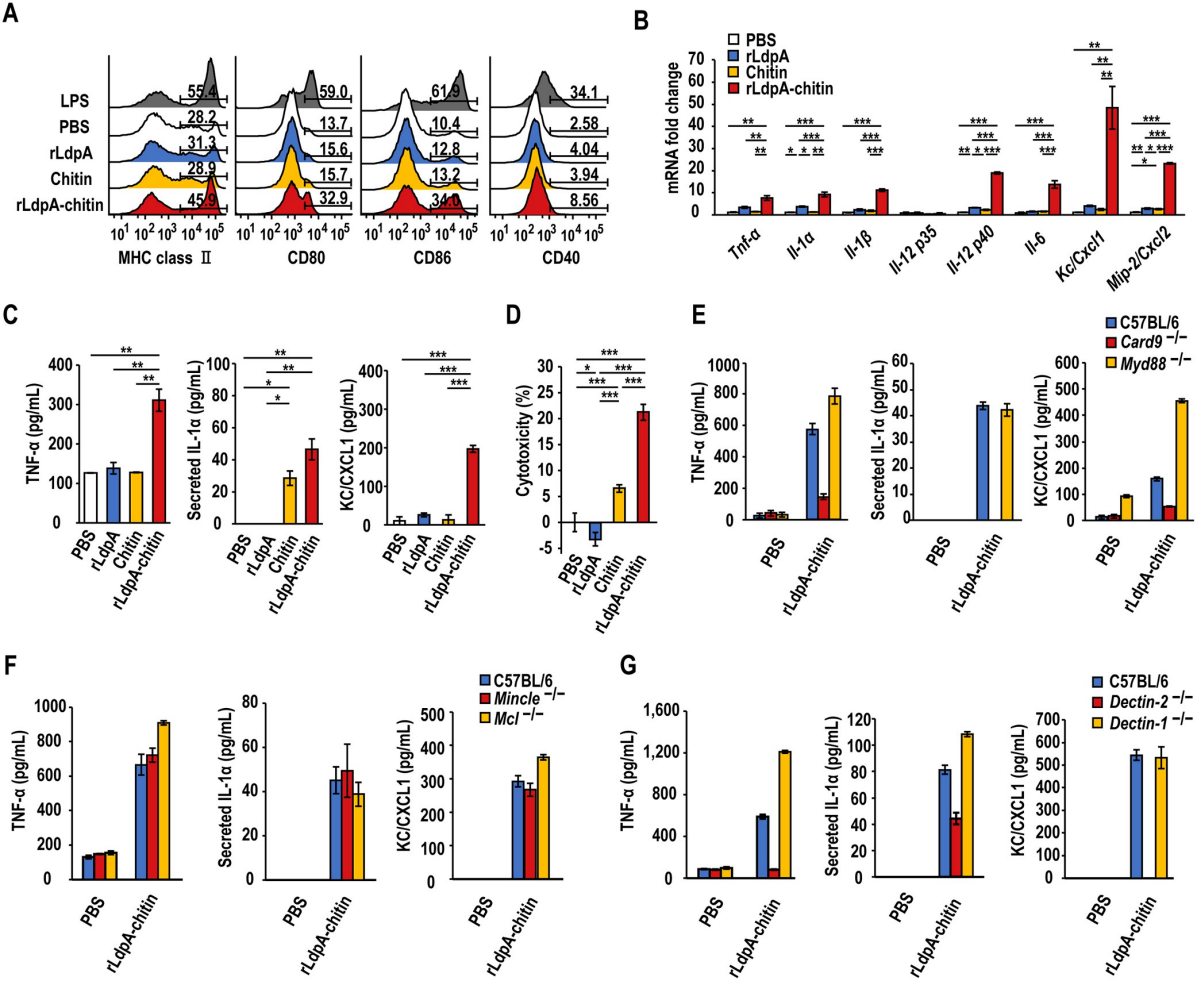
rLdpA activates BMDCs synergistically with chitin. (A) Bone marrow-derived dendritic cells (BMDCs) from C57BL/6 mice were incubated with recombinant LdpA (rLdpA) (1 μg/mL), chitin (50 μg/mL), rLdpA–chitin complex (rLdpA, 1 μg/mL; chitin, 50 μg/mL), lipopolysaccharide (LPS) (100 ng/mL), and phosphate-buffered saline (PBS) for 24 h. MHC class II, CD80, CD86, and CD40 cell-surface expression were measured by flow cytometry. (B) Proinflammatory cytokine and chemokine mRNA expression. After BMDCs derived from C57BL/6 mice were incubated with rLdpA (25 μg/mL), chitin (250 μg/mL), rLdpA–chitin complex (rLdpA, 1 μg/mL; chitin, 50 μg/mL), and PBS for 1.5 h, mRNA expression levels of *Tnf-α*, *Il-1α*, *Il-1β*, *Il-12 p35*, *Il-12 p40*, *Il-6*, *Kc/Cxcl1*, and *Mip-2/Cxcl2* were measured by quantitative real-time PCR. mRNA expression levels were normalized relative to *Gapdh* mRNA levels and are shown as fold change relative to the control PBS group. (C, D) BMDCs derived from C57BL/6 mice were incubated with rLdpA (1 μg/mL), chitin (50 μg/mL), rLdpA–chitin complex (rLdpA, 1 μg/mL; chitin, 50 μg/mL), and PBS for 24 h, TNF-α, IL-1α, and KC/CXCL1 protein levels in the culture supernatant were measured by enzyme-linked immunosorbent assay (ELISA) (C). Cytotoxicity was assessed by measuring the release of lactate dehydrogenase (LDH) (D). (E, F, G) BMDCs derived from C57BL/6, *Card9*^−/−^, *Myd88*^−/−^ (E), *Mincle*^−/−^, *MCL*^−/−^ (F), *Dectin-2*^−/−^ and *Dectin-1*^−/−^ (G) mice were incubated with rLdpA–chitin complex (rLdpA, 1 μg/mL; chitin, 50 μg/mL) and PBS for 24 h, and TNF-α, IL-1α, and KC/CXCL1 protein levels in the culture supernatant were measured by ELISA. Data are shown as the mean ± standard deviation (SD). *P < 0.05, **P < 0.01, and ***P < 0.001 by one-way ANOVA with *post hoc* Tukey–Kramer test (B, C, D).

We next examined whether rLdpA–chitin complex activated BMDCs. Incubation with rLdpA–chitin complex resulted in upregulation of the cell-surface expression of major histocompatibility complex (MHC) class II, CD80, CD86, and CD40 (Fig 4A). In addition, rLdpA–chitin complex markedly enhanced the mRNA expression levels of the proinflammatory cytokines, *Tnf-α*, *Il-1α*, *Il-1β*, *Il-6*, and *Il-12 p40*, and the chemokines, *Kc/Cxcl1* and *Mip-2/Cxcl2* (Fig 4B), as well as increasing the TNF-α, IL-1α, and KC/CXCL1 protein levels (Fig 4C). Furthermore, rLdpA–chitin complex increased chitin particle-induced cell death (Fig 4D). The rLdpA–chitin complex-mediated induction of proinflammatory cytokines and chemokines was dependent on caspase recruitment domain-containing protein 9 (Card9) and Dectin-2, and independent of myeloid differentiation marker 88 (Myd88), macrophage-inducible C-type lectin (Mincle), macrophage C-type lectin (MCL), and Dectin-1 (Fig 4E, 4F and 4G). Taken together, these observations indicated that the rLdpA–Dectin-2 interaction is crucial for the rLdpA–chitin complex-mediated activation of BMDCs.

### rLdpA–chitin complex induces Dectin-2-dependent neutrophilic airway inflammation

**Fig 5:**
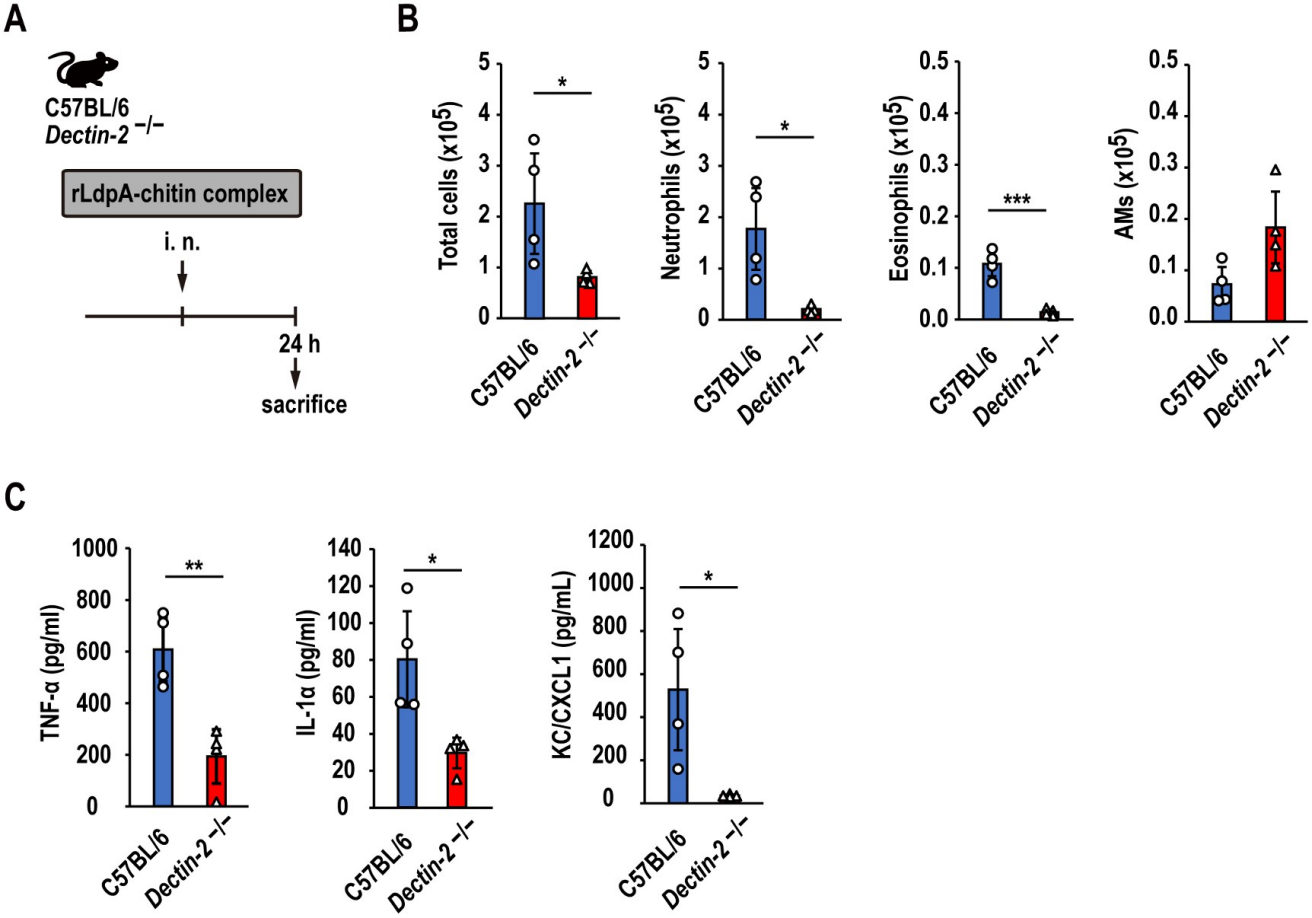
rLdpA–chitin complex induces Dectin-2-dependent neutrophilic airway inflammation. (A) A single dose of recombinant LdpA–chitin complex (rLdpA, 10 μg; chitin, 100 μg) was administered intranasally to C57BL/6 and *Dectin-2*^−/−^ mice. All animals were sacrificed at 24 h after administration and BAL fluids were collected. (B) The numbers of total cells, neutrophils, eosinophils, and alveolar macrophages (AMs) in the BAL fluid were measured by flow cytometry. (C) The levels of TNF-α, IL-1α, and KC/CXCL1 in the BAL fluid were measured by ELISA. Data are shown as the mean ± standard deviation (SD). (*n* = 4 mice/group). Each symbol represents an individual sample. *P* < 0.05, **P < 0.01, and ***P < 0.001 by unpaired two-tailed Student’s *t* test.

To investigate the effects of rLdpA–chitin complex on Dectin-2-induced airway inflammation *in vivo*, a single dose of rLdpA–chitin complex was administered intranasally to C57BL/6 and *Dectin-2*^−/−^ mice. All animals were sacrificed at 24 h after administration and BAL fluids were collected (Fig 5A). Compared to C57BL/6 mice, *Dectin-2*^−/−^ mice had significantly reduced levels of total cells, neutrophils, and eosinophils infiltration in the airways (Fig 5B). In contrast, in *Dectin-2*^−/−^ mice, the decrease in alveolar macrophages was suppressed compared to C57BL/6 mice (Fig 5B). A single dose of nasal administration of rLdpA–chitin complex also induced the levels of TNF-α, IL-1α, and KC/CXCL1 in the BAL fluid in a Dectin-2-dependent manner (Fig 5C). These observations suggested that rLdpA–chitin complex induces Dectin-2-dependent neutrophilic airway inflammation.

### rLdpA and chitin synergistically induce Th2 airway inflammation

**Fig 6:**
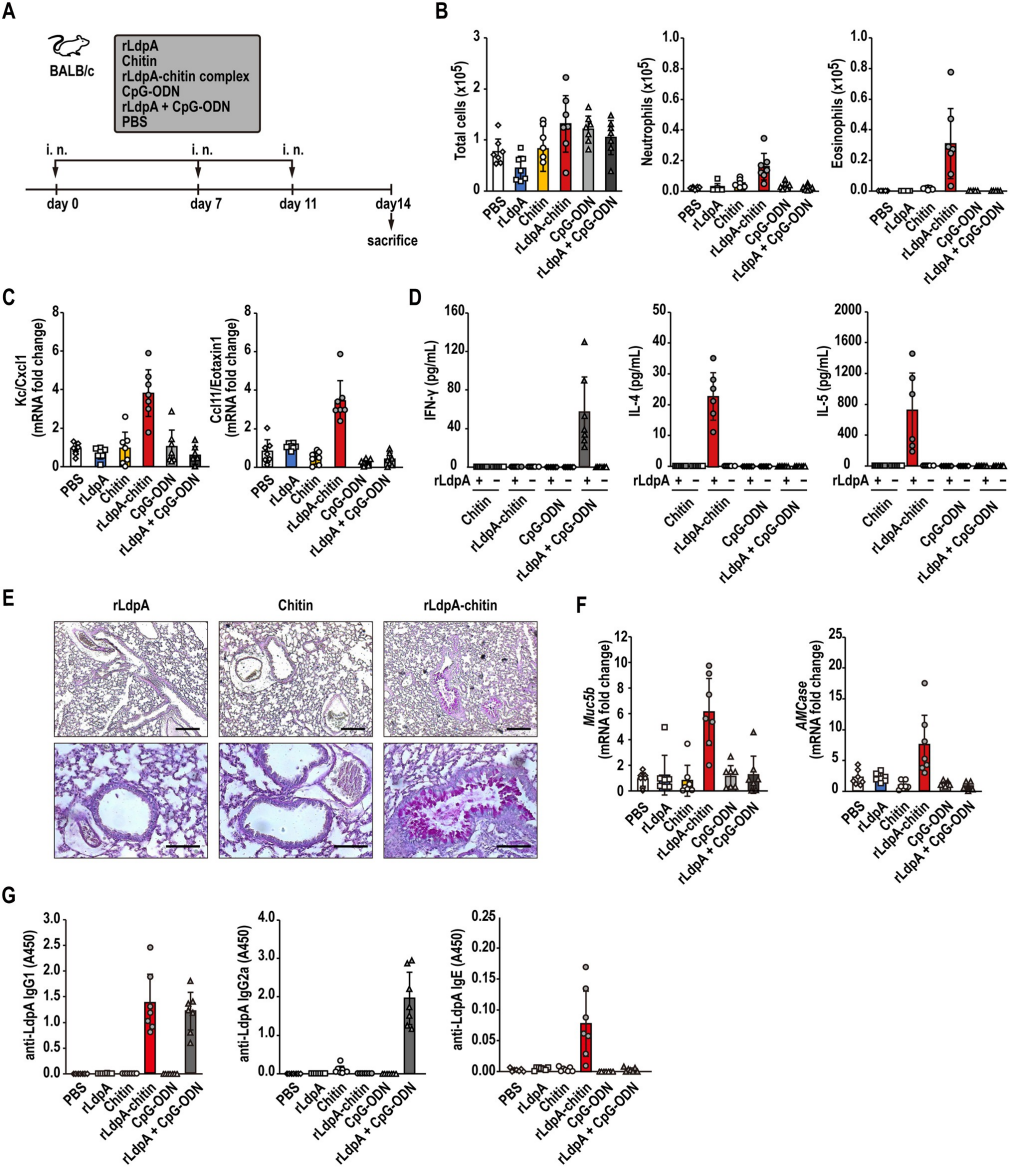
Nasal immunization with rLdpA-chitin complex induced a Th2 immune response. (A) rLdpA (10 μg), chitin (100 μg), rLdpA–chitin complex (rLdpA, 10 μg; chitin, 100 μg), CpG-ODN (20 μg), rLdpA+CpG-ODN (rLdpA, 10 μg; CpG-ODN, 20 μg), or vehicle (PBS) was administered intranasally to BALB/c mice three times. All animals were sacrificed at 72 h after the last nasal administration, and samples were obtained. (B) The numbers of total cells, neutrophils (CD11c^−^ Siglec F^−^ CD11b^+^ Ly-6G^+^), and eosinophils (CD11c^−^ Siglec F^+^) in bronchoalveolar lavage (BAL) fluid were measured by flow cytometry. (C) mRNA expression levels of *Kc/Cxcl1* and *Ccl11/Eotaxin-1* in the lung were measured by quantitative real-time PCR (qRT-PCR). The mRNA levels of these genes were normalized relative to *Gapdh* mRNA. The fold differences in expression are shown relative to the vehicle control group. (D) IL-4, IL-5, and IFN-γ secretion by mediastinal lymph node (MLN) cells after *ex vivo* re-stimulation with or without 10 μg/mL rLdpA. (E) Histological examination of lung tissues stained with Periodic acid–Schiff (PAS). Scale bar, 100 μm. (F) mRNA expression levels of *Muc5b* and *AMCase* in the lung were measured by qRT-PCR. The mRNA levels of these genes were normalized relative to *Gapdh* mRNA. The fold differences in expression are shown relative to the vehicle control group. (G) Levels of serum LdpA-specific IgG1, IgG2a, and IgE measured by indirect ELISA. Data are shown as the mean ± standard deviation (SD) (*n* = 6–7 mice/group). Each symbol represents an individual sample.

To investigate the synergistic effects of rLdpA and chitin particles on the airway immune response *in vivo*, BALB/c mice were intranasally administered rLdpA–chitin complex, rLdpA, chitin particles, CpG-ODN, or rLdpA+CpG-ODN three times (Fig 6A). In the group administered rLdpA–chitin complex, the level of eosinophil and neutrophils infiltration into the airway increased markedly (Fig 6B). Nasal administration of rLdpA–chitin complex also induced the expression of the eosinophil chemotactic protein, CCL11/Eotaxin-1, and neutrophil chemotactic protein, KC/CXCL1 (Fig 6C). We further examined the Th1/Th2 adaptive immune responses associated with rLdpA–chitin complex. The levels of the Th2-related cytokines, IL-4 and IL-5, were elevated in the culture supernatant of mediastinal lymph node cells from mice administered rLdpA–chitin complex after *ex vivo* re-stimulation with rLdpA, while no increase was observed in level of the Th1-related cytokine, INF-γ (Fig 6D). In contrast, INF-γ level was elevated in the mice administered rLdpA+CpG-ODN (Fig 6D). Histological examination of lung tissues showed that rLdpA–chitin complex induced hyperplasia of the mucin-secreting airway goblet cells, which play a role in the airway remodeling process in asthma (Fig 6E). mRNA expression levels of *Muc5b* and *AMCase* in the lung were elevated in the group administered rLdpA–chitin complex (Fig 6F). Serum LdpA-specific IgG1 and IgE levels were elevated in the group administered rLdpA–chitin complex (Fig 6G). In addition, rLdpA–chitin complex-induced Th2-driven airway inflammation was also observed in C57BL/6 mice, which are known to show differences in immune responsiveness to allergens in comparison to BALB/c mice (S4 Fig). These observations suggested that rLdpA and chitin induce Th2 airway inflammation in a synergistic manner.

### Dectin-2 deficiency attenuates the rLdpA–chitin complex-induced immune response

**Fig 7:**
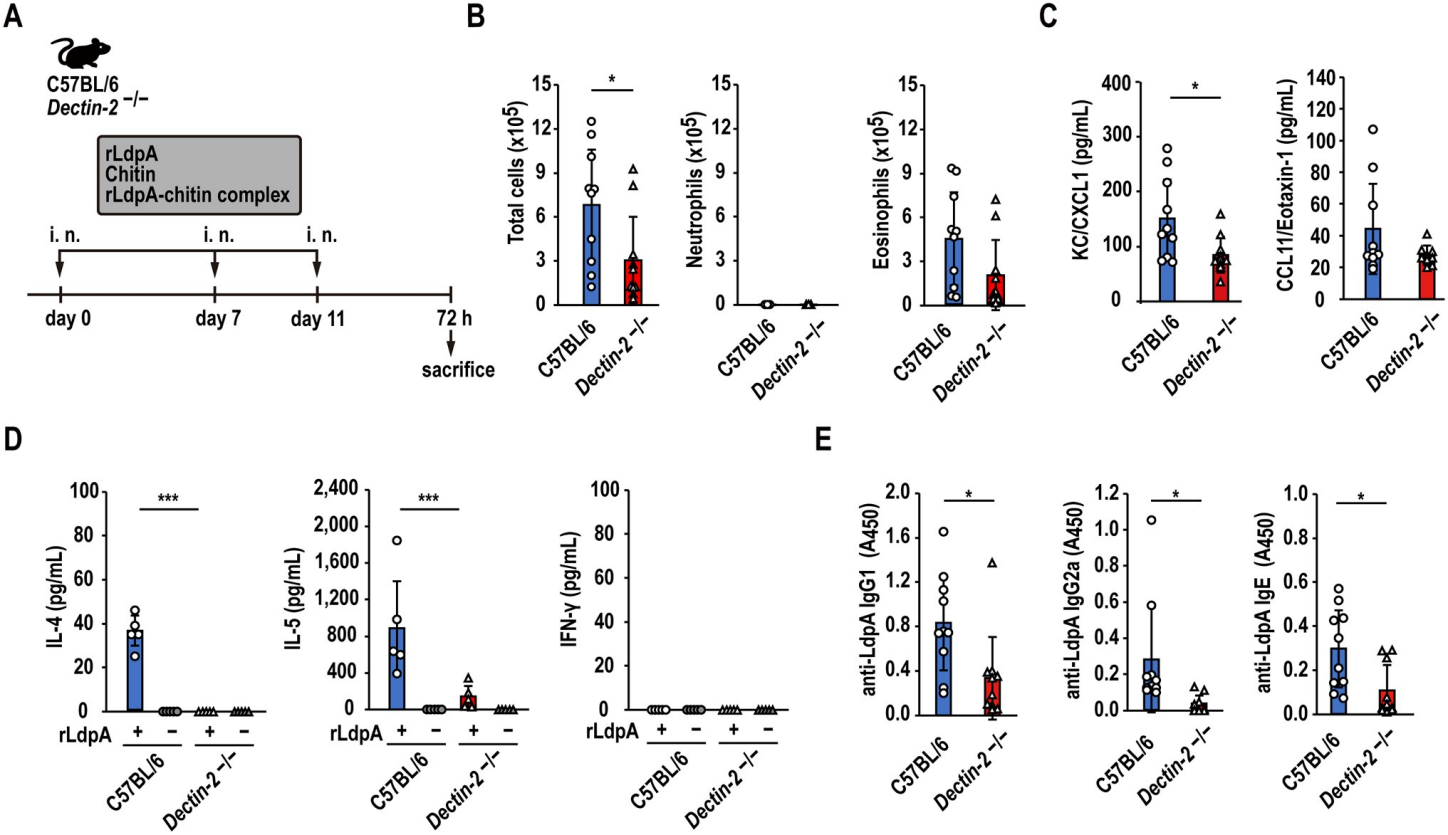
Deficiency of Dectin-2 attenuates the rLdpA–chitin complex-induced immune response. (A) Recombinant LdpA–chitin complex (rLdpA, 10 μg; chitin, 100 μg) were administered intranasally to *Dectin-2*^−/−^ and C57BL/6 mice three times. All animals were sacrificed 72 h after the last nasal administration, and samples were obtained. (B) The numbers of total cells, neutrophils, and eosinophils in BAL fluid were measured by flow cytometry. (C) KC/CXCL1 and CCL11/Eotaxin-1 protein levels in the BAL fluid were measured by ELISA. (D) IFN-γ, IL-4, and IL-5 secretion by spleen cells after *ex vivo* re-stimulation with or without 10 μg/mL rLdpA. (E) Levels of serum LdpA-specific IgG1, IgG2a, and IgE measured by indirect ELISA. Data are shown as the mean ± standard deviation (SD) (*n* = 5–10 mice/group). Each symbol represents an individual sample. *P < 0.05, **P < 0.01, and ***P < 0.001 by unpaired two-tailed Student’s *t* test.

The infiltration of eosinophils into the airway (Fig 7B), KC/CXCL1 and CCL11/Eotaxin-1 levels in BAL fluid (Fig 7C), IL-4 and IL-5 levels in the spleen cell culture supernatant after *ex vivo* re-stimulation with rLdpA (Fig 7D), and serum LdpA-specific IgG1, IgG2a, and IgE levels (Fig 7E) were attenuated in *Dectin-2*^−/−^ mice administered rLdpA–chitin complex in comparison to C57BL/6 mice. These observations indicated that Dectin-2 is crucial for the LdpA–chitin complex-induced immune response.

### Serum LdpA-specific immunoglobulin levels are elevated in pulmonary aspergillosis patients

**Fig 8:**
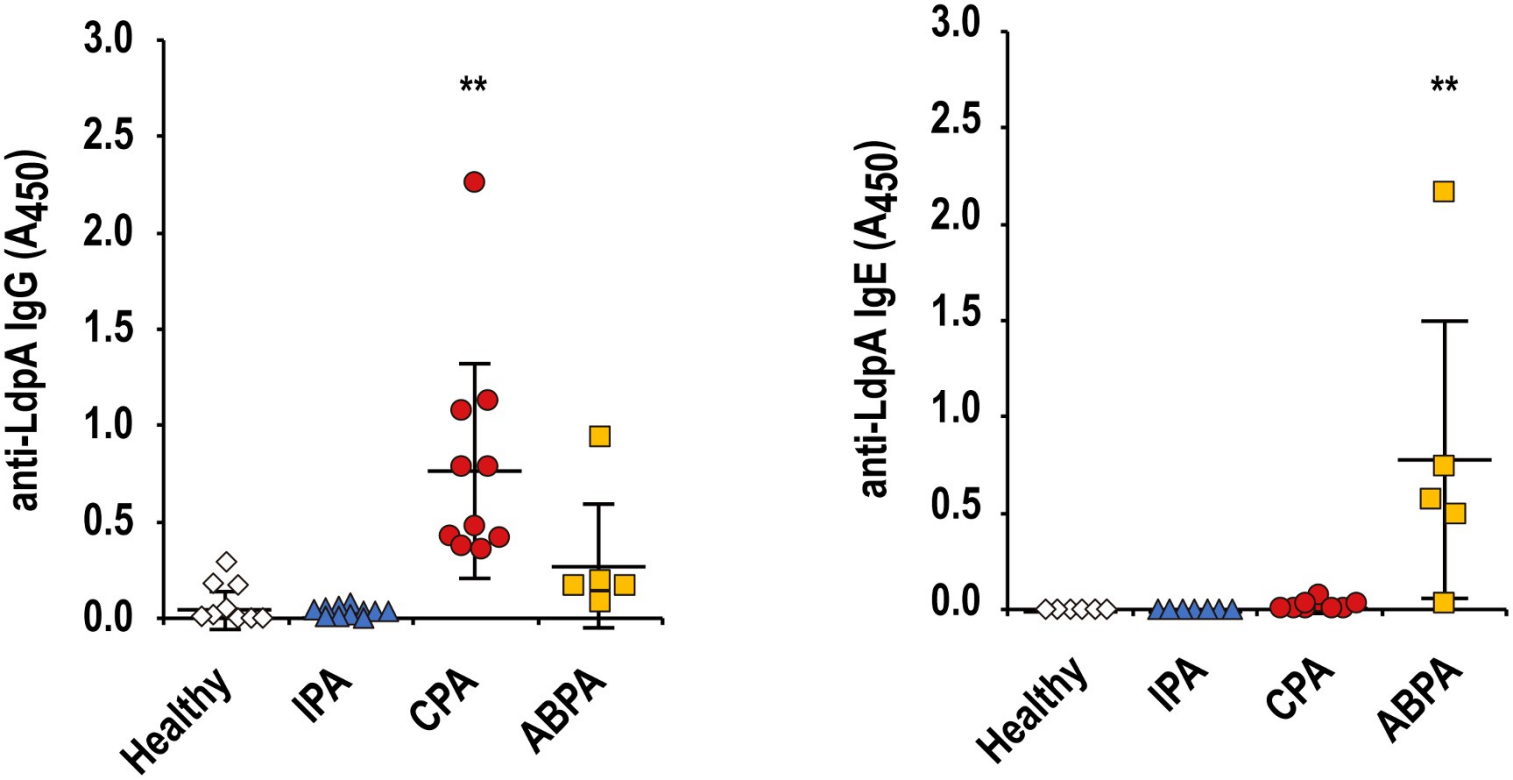
Serum LdpA-specific immunoglobulin levels are elevated in patients with pulmonary aspergillosis. Serum LdpA-specific IgG and IgE levels in invasive pulmonary aspergillosis (IPA), chronic pulmonary aspergillosis (CPA), and allergic bronchopulmonary aspergillosis (ABPA) patients and healthy individuals were measured by indirect enzyme-linked immunosorbent assay (ELISA). Data are shown as the mean ± standard deviation (SD) (IPA patients, *n* = 11; CPA patients, *n* = 10; ABPM patients, *n* = 5; healthy individuals, *n* = 10). Each symbol represents an individual sample. **P < 0.01 by one-way ANOVA with Dunnett’s multiple comparison test.

We next investigated the serum LdpA-specific immunoglobulin (Ig) levels in patients with three types of pulmonary aspergillosis, *i*.*e*., invasive pulmonary aspergillosis (IPA), chronic pulmonary aspergillosis (CPA), and allergic bronchopulmonary aspergillosis (ABPA), by indirect enzyme-linked immunosorbent assay (ELISA). The demographic characteristics of the participants and healthy volunteers are shown in S1 Table. The serum levels of LdpA-specific IgG and IgE were elevated in patients with CPA and ABPA, respectively, compared with healthy controls and patients with IPA (Fig 8). These observations suggested that LdpA is recognized as an antigen in patients with chronic and allergic lung *A*. *fumigatus* infection.

## Discussion

The outer cell walls of yeasts, such as *Candida* and *Saccharomyces* species, contain heavily mannosylated glycoproteins, known as mannoproteins [39,40]. Mannoproteins comprise up to 40% of the cell wall dry weight of the human pathogenic yeast, *Candida albicans* [40]. In contrast to yeast, mannose chains are components of cell wall galactomannan in the filamentous fungus *A*. *fumigatus*, which are bound covalently to the inner cell wall of the β-(1,3)-glucan–chitin core [39,41]. In addition, the *A*. *fumigatus* cell wall contains mannose chains as glycans of *O*-linked glycoproteins and *N*-linked glycoproteins [42]. The structures of *O*-glycans on glycoprotein are quite diverse between different organisms and even within fungal species [43]. Trimble *et al*. reported that *O*-glycans in the recombinant human bile salt-stimulated lipase expressed in *P*. *pastoris* possessed a linear chain of up to six mannose residues, including Man1-*O*, Manα1-2Man1-*O*, and Manα1-2Manα1-2Man1-*O* [44]. In their study, the most abundant *O*-glycan was Manα1-2Man1-*O* followed by Manα1-2Manα1-2Man1-*O* [44]. Consistent with previous reports, recombinant *A*. *fumigatus* LdpA expressed in yeast *P*. *pastoris* was an *O*-linked glycoprotein with terminal α-mannose residues. In contrast to *P*. *pastoris*, the *O*-glycans in the glycoproteins of *A*. *fumigatus* have complex structures, but the *O*-glycans of *A*. *fumigatus* include Man1-*O* and Manα1-2Man1-*O* [45]. These reports suggest that the *A*. *fumigatus* native LdpA could show immune responses similar to rLdpA expressed in *P*. *pastoris*.

Ishikawa *et al*. reported that *O*-linked mannoprotein W1 isolated from the pathogenic fungus, *Malassezia*, was a Dectin-2 ligand, and the α-1,2-mannosyl residues of W1 were necessary and sufficient for recognition by Dectin-2 [37]. Recently, Reedy *et al*. reported that Dectin-2 is a receptor for *A*. *fumigatus* galactomannan [46]. In the present study, we showed that rLdpA expressed in *P*. *pastoris* was a ligand for Dectin-2, and we speculated that the native LdpA may be a strong candidate for the Dectin-2 ligand of *A*. *fumigatus*.

Lectins involved in innate immunity as pattern recognition receptors (PRRs) have been proposed to bind foreign pathogens not only by binding specificity but also by density-dependent recognition of surface glycans [47,48]. The addition of rLdpA to the medium did not transmit the signal to Dectin-2-NFAT-GFP reporter cells, but rLdpA–Dectin-2 signals were transmitted when rLdpA was immobilized on the plate. The induction of inflammatory cytokines by Dectin-2 signaling in BMDCs required stimulation with high concentrations of rLdpA. In contrast, low concentrations of rLdpA bound to chitin particles strongly induced phagocytosis and the expression of both cytokines and chemokines, suggesting that the high density of rLdpA bound on the surface of chitin particles transmitted the signal to Dectin-2.

Dectin-2 was reported to promote house dust mite-induced Th2-type cell differentiation and allergic airway inflammation in mice [30,31,49], but there have been no previous studies regarding the involvement of Dectin-2 in fungal-induced allergic airway inflammation. Here, we showed that rLdpA–chitin complex induced Th2-type adaptive immune responses, which was attenuated by Dectin-2 deficiency *in vivo*. Moreover, the levels of serum LdpA-specific IgE were elevated in patients with ABPA. These observations strongly suggested that native LdpA is involved in *A*. *fumigatus*-induced allergic immune responses.

Chitin has been shown to exert size-dependent effects on innate immune responses [12]. The production of TNF-α in macrophages *in vitro* is induced by phagocytable-size chitin particles but not by large non-phagocytable-size chitin particles [13–18]. In contrast to these findings, our *in vitro* results showed that small chitin particles (0.6–5.9 μm) did not induce TNF-α production in BMDCs. Low-purity chitin was suggested to potentially lead to misinterpretation of experimental results because commercially available chitin contains trace amounts of glucose and protein and several other unidentified contaminants [50]. However, many studies to date did not describe the methods used for purification or the chitin particle size, thus making it difficult to interpret the immune response induced by chitin.

In the present study, we showed that the phagocytosis of chitin particles by BMDCs induced IL-1α production and cell death-mediated IL-1α release. Moreover, we found that intranasal administration of chitin particles induced IL-1α-mediated acute neutrophilic airway inflammation *in vivo*, and this chitin particle-induced acute neutrophilic airway inflammation was attenuated by pretreatment with anti-IL-1α neutralizing antibody. IL-1α has been shown to be involved in activation of the innate immune system and induction of inflammation [38,51]. Some studies showed that IL-1α is rapidly released from pre-existing stocks in alveolar macrophages and promotes subsequent lung inflammation through the stimulation of IL-1β production in response to fine particle exposure in the mouse airway [52,53]. IL-1α and IL-1β show the same functions by sharing a common receptor, IL-1 type 1 receptor (IL-1R1) [54]. Takeshige *et al*. reported that inhaled chitin induces airway inflammation, airway hyperresponsiveness, and IL-1β production [55]. In addition to activating innate immunity, IL-1 (IL-1α and IL-1β) is known to have an adjuvant effect involved in activating the adaptive immune system [27,52,56]. Arae *et al*. reported that chitin- and IL-33-stimulated dendritic cell-derived IL-1β promoted OVA-specific Th2 cell activation, thus aggravating OVA-induced airway inflammation [27]. Kuroda *et al*. reported that the intratracheal instillation of IL-1α plus OVA induced strong OVA-specific IgE responses [52]. Bueter *et al*. reported that chitin fails to activate inflammasomes and induce mature IL-1β secretion in LPS-primed bone marrow-derived macrophages (BMMs) [57]. Consistent with their data, rLdpA and rLdpA-chitin complex induced IL-1β mRNA expression in BMDCs but failed to induce secretion of mature IL-1β (S5 Fig), suggesting that chitin failed to activate inflammasomes. Based on these previous reports and our data, we speculated that phagocytosis of chitin particles by antigen-presenting cells may induce cell death-mediated release of IL-1α, which subsequently plays an essential role in allergic airway inflammation induced by rLdpA-chitin complex. However, this study had the limitation that *in vivo* experiments did not demonstrate whether IL-1α is involved in the induction of Th2 immune responses by the rLdpA-chitin complex. This issue should be addressed in the future.

De Silva *et al*. reported that the adjuvant properties of chitin are mediated by a pathway(s) involving and regulated by TLR2, MYD88, and IL-17A [22,58]. Fuchs *et al*. reported that chitin oligomer binds directly to TLR2 and triggers inflammation in an oligomer size-dependent manner [59]. Their study suggested that contamination of chitin particles with chitin oligomers or the presence of host chitinases, such as Chitotriosidase and Acidic mammalian chitinase (AMCase) may affect the experimental results.

In conclusion, the results of the present study showed that *A*. *fumigatus* rLdpA expressed in *P*. *pastoris* is an *O*-linked glycoprotein containing the terminal α-mannose residues recognized by the host CLR, Dectin-2. Chitin particles induced acute neutrophilic airway inflammation mediated by cell death-associated IL-1α release. rLdpA potently induced Th2-driven allergic airway inflammation synergistically with chitin. Therefore, this study provided new insights into fungal-induced allergic airway diseases.

## Materials and methods

### Ethics statement

Serum samples obtained from each patient and healthy volunteers were collected with written, informed consent, and all experimental procedures were approved by the Research Ethics Committee of the Graduate School of Medicine, Chiba University (approval number: 3237). All animal experiments were approved by the Committee on Animal Experiments of Chiba University (approval numbers: 2–326, 2–327, 3–272) and carried out in accordance with the Chiba University Animal Experimentation Regulations.

### Animals

Specific pathogen-free (SPF) male C57BL/6 and BALB/c mice aged 5–7 weeks old were purchased from Charles River Laboratories Japan. Dectin-2- and Dectin-1-deficient mice in the C57BL/6 background were described previously [28,60]. Mincle- and MCL-deficient mice in the C57BL/6 background were described previously [61,62]. Myd88-deficient mice in the C57BL/6 background [63] were purchased from Oriental Bioservice (Kyoto, Japan). Card9-deficient mice in the C57BL/6 background were described previously [64]. All mice were housed under SPF conditions with food and water available *ad libitum*.

### Fungi

S2 Table shows the *P*. *pastoris* strains used in this study. *P*. *pastoris* were maintained on potato dextrose agar (PDA) (BD Biosciences, Franklin Lakes, NJ, USA) at 25°C.

### LdpA-specific Igs

The 96-well plates (Nunc MaxiSorp; Thermo Fisher Scientific, Waltham, MA, USA) were coated with rLdpA (0.2 μg/mL in 0.05 M sodium carbonate buffer, pH 9.6) and incubated overnight at 4°C. The rLdpA protein in each well was then blocked with phosphate-buffered saline (PBS) (0.2 g/L potassium chloride, 0.2 g/L potassium dihydrogen phosphate, 8 g/L sodium chloride, 1.15 g/L disodium hydrogen phosphate, pH 7.4) containing 2% BSA. Dilutions of human serum samples (1:200 and 1:10) were prepared for IgG and IgE, respectively. Dilutions of mouse serum samples (1:65,536, 1:512, and 1: 10) were prepared for IgG1, IgG2a, and IgE, respectively. The LdpA-specific absorbance values were determined with biotinylated anti-human IgG monoclonal antibody (mAb) (G18-145; BD Biosciences), biotinylated anti-human IgE mAb (f0822; Biomatrix Research, Chiba, Japan), biotinylated anti-mouse IgG1 mAb (A85-1; BD Pharmingen, San Diego, CA, USA), biotinylated anti-mouse IgG2a mAb (R19-15; BD Pharmingen), and biotinylated anti-mouse IgE mAb (R35-72; BD Pharmingen). Biotinylated mAbs were detected with Pierce High Sensitivity Streptavidin-Horseradish peroxidase (HRP) (Thermo Fisher Scientific, Waltham, MA, USA) and tetramethylbenzidine substrate (Bio-Rad, Hercules, CA, USA). The reaction was quenched using 1 *N* sulfuric acid, and absorbance was measured at 490 nm (A_490_) using an automated plate reader (Sunrise Rainbow Thermo RC; Tekan, Männedorf, Switzerland).

### Recombinant LdpA

The *A*. *fumigatus ldpA* CDS lacking the signal sequence was amplified by PCR from pCR2.1-*ldpA* [33]. The PCR product was cloned into the pPICZαC vector (Thermo Fisher Scientific) at the N-terminus of the c-Myc-tag and 6×His-tag using an In-Fusion HD Cloning Kit (Clontech Laboratories). The resulting plasmid, pPICZαC-*ldpA*, was used to transform *P*. *pastoris* GS115 (Thermo Fisher Scientific) by electroporation. The secretion of rLdpA into the culture supernatant was induced by addition of methanol. After removing *P*. *pastoris* cells, rLdpA in the culture supernatant was purified using Ni Sepharose 6 Fast Flow (GE Healthcare, Wauwatosa, WI, USA). After buffer exchange to PBS, rLdpA was concentrated using Amicon Ultra-15 Centrifugal Filter Units with Ultracel-10 membrane (Merck Millipore, Billerica, MA, USA). The final protein concentration was determined using a Pierce BCA Protein Assay Kit (Thermo Fisher Scientific) with BSA as the standard. Purified rLdpA was confirmed by SDS-PAGE and Western blotting analysis using anti-c-Myc mAb (9E10; Sigma-Aldrich, St. Louis, MO, USA) and HRP-conjugated anti-mouse IgG secondary antibody (A4416; Sigma-Aldrich). The plasmids used in this study are summarized in S3 Table.

### Affinity of rLdpA for insoluble polysaccharides

The affinity of rLdpA for insoluble polysaccharides was determined by incubating 10 μg of rLdpA with 1 mg each of chitin (C7170; Sigma-Aldrich), chitin beads (New England Biolabs, Ipswich, MA, USA), chitosan (≥ 80.0% deacetylation) (FUJIFILM Wako Pure Chemical), and cellulose (FUJIFILM Wako Pure Chemical) in PBS (total volume = 100 μL) at 37°C for 30 min, with gentle agitation. The insoluble fraction was pelleted by centrifugation, and the supernatant was collected. The insoluble fraction was washed three times with PBS and resuspended in distilled water. rLdpA in the pellet and supernatant was evaluated by SDS-PAGE, and the gel was stained with Coomassie Brilliant Blue (FUJIFILM Wako Pure Chemical, Osaka, Japan). BSA (FUJIFILM Wako Pure Chemical) was used as a control to determine nonspecific binding.

### Glycoprotein staining

rLdpA protein was transferred onto polyvinylidene difluoride (PVDF) membranes (Merck Millipore) after SDS-PAGE. The membranes were then stained with periodic acid–Schiff (PAS) using a Glycoprotein Western Detection Kit (Takara Bio, Kyoto, Japan), according to the manufacturer’s instructions.

### Chemical deglycosylation

Chemical deglycosylation of rLdpA was performed using trifluoromethanesulfonic acid (TFMS) (Sigma-Aldrich), as described previously [35,36]. After treatment with TFMS, the shift in protein M_r_ was assessed by SDS-PAGE. Bovine fetuin (Sigma-Aldrich) and BSA (Sigma-Aldrich) were used as glycoprotein and non-glycoprotein controls, respectively.

### Demannosylation

The rLdpA was incubated with α1–2, -3, and -6 mannosidase (New England Biolabs) at 37°C for 1 h and the shift in protein M_r_ was examined by SDS-PAGE, followed by Western blotting with anti-c-Myc mAb (9E10; Sigma-Aldrich) and HRP-conjugated anti-mouse IgG secondary antibody (A4416; Sigma-Aldrich).

### Lectin binding assay

Aliquots of 20 μg of rLdpA were reacted with 1 mg of Dynabeads (Dynabeads His-Tag Isolation & Pulldown kit; Thermo Fisher Scientific) to generate rLdpA–Dynabeads complex. After incubation of rLdpA–Dynabeads complex with fluorescein isothiocyanate (FITC)-labeled concanavalin A (ConA) (20 μg) (Sigma-Aldrich), FITC-labeled wheatgerm agglutinin (WGA) (20 μg) (Sigma-Aldrich), and FITC-labeled peanut agglutinin (PNA) (20 μg) (Sigma-Aldrich), lectins bound to rLdpA were measured by flow cytometry.

### Dectin-2-NFAT-GFP reporter assay

The 96-well plates (Nunc MaxiSorp; Thermo Fisher Scientific) were coated with rLdpA (0.1, 1, and 10 μg/mL in 0.05 M sodium carbonate buffer, pH 9.6) and incubated overnight at 4°C. After washing with PBS three times, NFAT-GFP reporter cells expressing FcRγ with or without Dectin-2 [65] were cultured for 24 h. The percentage of GFP-positive cells was measured by flow cytometry (BD FACSVerse; BD Biosciences). Data were analyzed using FlowJo version 10 (BD Biosciences).

### Gene expression analysis by qRT-PCR

Total RNA was isolated using RNAiso plus (Takara Bio) and Direct-zol RNA MiniPrep (Zymo Research, Orange, CA, USA), and treated with DNase using a Turbo DNA-free Kit (Thermo Fisher Scientific) to remove DNA contamination. Subsequently, cDNA was synthesized using a PrimeScript RT Master Mix (Takara Bio), according to the manufacturer’s instructions. Quantitative real-time PCR (qRT-PCR) was performed on an Applied Biosystems StepOnePlus Real-Time PCR System (Thermo Fisher Scientific) using TB Green Fast qPCR Mix (Takara Bio) and gene-specific primers (S4 Table). Gene expression levels were normalized relative to *Gapdh* mRNA levels and are shown as fold change relative to the vehicle control group.

### Chitin purification

Chitin was purified as described by Roy *et al*. [66]. Briefly, chitin (C7170; Sigma-Aldrich) was dissolved in 12.5 M HCl and incubated at 40°C for 30 min. The solution was transferred to a cooled beaker and then slowly neutralized with ice-cold sodium hydroxide (NaOH). The insoluble fraction was collected by centrifugation at 1000 × *g* for 30 s, and washed three times with water and once with ethanol. The purified chitin particles were filtered through a 10-μm nylon filter. The filtrate was centrifuged, and the sediment was dried in a desiccator. Chitin particle size was evaluated by flow cytometry. Chitin particles were sonicated before use in the experiments.

### Preparation of rLdpA–chitin complex

The rLdpA–chitin complex was generated by mixing 20 μg of rLdpA with 1 mg of chitin for *in vitro* experiments and 100 μg of rLdpA with 1 mg of chitin for *in vivo* experiments. Each mixture was incubated at room temperature for 30 min.

### Fluorescent labeling of rLdpA and chitin

rLdpA was labeled with FITC isomer-I (Dojindo, Kumamoto, Japan, Kumamoto, Japan), according to the manufacturer’s instructions. Chitin was labeled with 25 μg/mL of calcofluor white (CFW) (Fluorescent Brightener 28; Sigma-Aldrich) at room temperature for 30 min, washed three times with PBS, and then resuspended in PBS. To generate rLdpA-FITC-chitin-CFW complex, 1 mg of CFW-labeled chitin was incubated with 20 μg of FITC-labeled rLdpA at room temperature for 30 min.

### Fluorescence microscopy

Fluorescently labeled samples were examined using a confocal laser scanning microscope (STELLARIS 5; Leica Microsystems, Wetzlar, Germany) and an inverted fluorescence microscope (BZ-9000; Keyence).

### Mouse BMDCs

Mouse bone marrow-derived dendritic cells (BMDCs) were obtained as described by Lutz *et al*. [67]. Briefly, mouse bone marrow was obtained from the tibiae and femora of mice. Bone marrow cells were cultured in RPMI 1640 (R8758; Sigma-Aldrich) supplemented with 100 U/mL GM-CSF (Peprotech, Rocky Hill, NJ, USA), 10% (v/v) heat-inactivated fetal bovine serum (FBS) (Thermo Fisher Scientific), 0.1% (v/v) 2-mercaptoethanol (Thermo Fisher Scientific), 1mM sodium pyruvate (Thermo Fisher Scientific), 1× MEM non-essential amino acids (Thermo Fisher Scientific), 100 U/mL penicillin, and 100 μg/mL streptomycin (FUJIFILM Wako Pure Chemical). Cultures were fed on days by 2, 4, and 6 replacing approximately 75% of the medium. At 6 ± 1 days, loosely adherent and non-adherent cells were harvested as BMDCs.

### Phagocytosis assay

Phagocytosis assay was performed by dispensing mouse BMDCs into 24-well plates at a concentration of 2.5 × 10^5^ cells/well and incubating with FITC- and CFW-labeled rLdpA–chitin complex (rLdpA, 1 μg/mL; chitin, 50 μg/mL), FITC-labeled rLdpA (1 μg/mL), or CFW-labeled chitin (50 μg/mL) at 37°C under an atmosphere of 5% CO_2_ for the indicated time. The mean fluorescence intensity (MFI) of cells was measured by flow cytometry (BD FACSVerse; BD Biosciences). Data were analyzed using FlowJo version 10 (BD Biosciences).

### Phagocytosis inhibition assay

Phagocytosis inhibition assay was performed by dispensing mouse BMDCs into 24-well plates at a concentration of 2.5 × 10^5^ cells/well and incubating with 1 mg/mL mannan from *Saccharomyces cerevisiae* (Sigma-Aldrich), 1 mg/mL d(+)-mannose (FUJIFILM Wako Pure Chemical), 1 mg/mL laminarin (Nacalai Tesque, Kyoto, Japan), 1 mg/mL dextran from *Leuconostoc* spp. (M_r_ 450000–650000; Sigma-Aldrich), 1 mg/mL d(+)-galactose (FUJIFILM Wako Pure Chemical), 1 mg/mL *N*-acetyl-d(+)-glucosamine (FUJIFILM Wako Pure Chemical), or PBS at 37°C under an atmosphere of 5% CO_2_ for 30 min, followed by addition of CFW-labeled rLdpA–chitin complex (rLdpA, 1 μg/mL, chitin, 50 μg/mL). After 4-h incubation, the MFI of rLdpA–chitin complex-phagocytosed cells was measured by flow cytometry (BD FACSVerse; BD Biosciences). Data were analyzed using FlowJo version 10 (BD Biosciences).

### Measurement of cytokines and chemokines

Cytokine and chemokine levels were determined using mouse TNF-α, IFN-γ, IL-1α, IL-1β, IL-4, IL-5, KC/CXCL1, Mip-2/CXCL2, and CCL11/Eotaxin1 ELISA kits (R&D Systems, Minneapolis, MN, USA).

### Expression of costimulatory molecules

Mouse BMDCs were dispensed into 12-well plates at a concentration of 5 × 10^5^ cells/well and incubated with rLdpA (1 μg/mL), chitin (50 μg/mL), rLdpA–chitin complex (rLdpA, 1 μg/mL; chitin, 50 μg/mL), or lipopolysaccharide (LPS, 10 ng/mL) (eBioscience, San Diego, CA, USA) for 16 h. Cells were suspended in FACS buffer (2% heat-inactivated FBS and 1 mM EDTA in PBS, pH 7.4). Fc receptors were blocked with anti-mouse CD16/32 mAb (93; eBioscience). Cells were stained with a mixture of fluorochrome-conjugated monoclonal antibodies (mAbs) to CD11c (N418; eBioscience), MHC class II (M5/114.15.2; Miltenyi Biotec), CD40 (FGK45.5; Miltenyi Biotec), CD80 (16-10A1; Miltenyi Biotec), and CD86 (PO3.3; Miltenyi Biotec) in FACS buffer. CD11c^+^ cells were gated, and MHC class II, CD40, CD80, and CD86 cell-surface expression were measured by flow cytometry (BD FACSVerse; BD Biosciences). Data were analyzed using FlowJo version 10 (BD Biosciences).

### Cytotoxicity assay

Cytotoxicity was analyzed by measuring lactate dehydrogenase (LDH) activity released from the damaged cells using a Cytotoxicity LDH Assay Kit-WST (Dojindo Laboratories).

### Animal model

Mice were anesthetized with isoflurane and intranasally administered 20 μL of PBS containing 10 μg of rLdpA (rLdpA group), 100 μg of chitin (chitin group), a mixture of 10 μg rLdpA and 100 μg chitin (rLdpA–chitin complex group), 20 μg of CpG-ODN (K3; GeneDesign, Osaka, Japan) (CpG-ODN group), a mixture of 10 μg of rLdpA and 20 μg of CpG-ODN (rLdpA+CpG-ODN group), or PBS as a control (vehicle control group). Anti-IL-1α mAb (ALF-161; eBioscience) or Armenian hamster IgG isotype control (eBio299Arm; eBioscience) was injected into the peritoneal cavity of mice at a dose of 80 μg/mouse 1 h before intranasal chitin administration [53]. Mice were euthanized by cardiac puncture under anesthesia with ketamine and xylazine at the time points indicated in the Figures.

### Total and differential leukocyte counts in BAL fluid

Airway contents were recovered by installation and retrieval of 0.7 mL of sterile PBS. The BAL fluid was centrifuged, and the cell pellet was resuspended in PBS. Cells were counted using a hemocytometer under a light microscope. To determine the differential leukocyte count, cells were resuspended in FACS buffer, and Fc receptors were blocked with anti-mouse CD16/32 mAb (93; eBioscience). Cells were stained with a mixture of fluorochrome-conjugated mAbs to CD11c (N418; eBioscience), CD11b (M1/70; eBioscience), Siglec F (E50-2440; BD Biosciences), and Ly-6G (1A8-Ly; eBioscience) in FACS buffer. After lysis of erythrocytes in RBC lysis buffer (eBioscience), cells were resuspended in FACS buffer. Samples were analyzed by flow cytometry (BD FACSVerse; BD Biosciences). Neutrophils were identified as CD11c^−^ Siglec F^−^ CD11b^+^ Ly-6G^+^ cells, eosinophils were identified as CD11c^−^ Siglec F^+^ cells and alveolar macrophages were identified as CD11c^+^ Siglec F^+^ cells. Data were analyzed using FlowJo version 10 (BD Biosciences).

### LdpA-specific re-stimulation of spleen and mediastinal lymph node cells

After airway exposure as described above, mouse spleen or mediastinal lymph node (MLN) cells were isolated and cultured in RPMI 1640 medium supplemented with 10% heat-inactivated FBS, 100 U/mL penicillin, and 100 μg/mL streptomycin, with or without rLdpA (10 μg/mL) at 37°C under an atmosphere of 5% CO_2_ for 72 h. Cell culture supernatants were collected for cytokine determination by ELISA.

### Lung histopathology

Mouse lungs were fixed in buffered 10% formalin. The fixed lungs were routinely embedded in paraffin and cut into sections 4–5 μm thick, which were stained with PAS.

### Quantification and statistical analysis

Statistical analyses were performed using GraphPad InStat 3 software (GraphPad Software, San Diego, CA, USA). In all analyses, *P* < 0.05 was taken to indicate statistical significance. Details of statistical tests and sample sizes are indicated in the figure legends. *P*-values are indicated in the figures.

## Supporting information

S1 Table Demographic Characteristics of the Participants and Healthy controls. (PDF)

S2 Table List of *Pichia pastoris* Strains Used in This Study. (PDF)

S3 Table Plasmids Used in This Study. (PDF)

S4 Table Primers Used for Quantitative RT-PCR in This Study. (PDF)

S1 Fig Expression of *Aspergillus fumigatus* LdpA in *Pichia pastoris*. (A) Total protein levels in the culture supernatant. *P*. *pastoris* GS115 and *ldpA* transformant GS115-LdpA were cultured in buffered methanol medium (BMM), and protein expression was induced by the addition of methanol. Total protein levels in the culture supernatant were measured by BCA protein assay. (B, C) Purified recombinant LdpA (rLdpA) was confirmed by SDS-PAGE (B) and Western blotting analysis using anti-c-Myc antibody and HRP-conjugated secondary antibody (C).
(TIF)

S2 Fig Phagocytosis of Dynabeads fails to induce cell death and IL-1α release. BMDCs from C57BL/6 mice were incubated with Dynabeads, chitin, or PBS for 24 h. (A) After washing with PBS, cells were stained with Alexa Fluor 555 Phalloidin (yellow), nuclei were stained with NucRed Live 647 ReadyProbes Reagent (red), and observed by confocal laser scanning microscopy. (B) Cytotoxicity was assessed by measuring the release of LDH. (C) IL-1α protein levels in the culture supernatant were measured by ELISA.
(TIF)

S3 Fig Chitin-binding capacity of rLdpA. After incubation of serially diluted rLdpA (2.5–40 μg) with chitin (1 mg) in 100 μL of PBS, rLdpA in the supernatant (sup.) and sediment (sed.) was evaluated by SDS-PAGE.
(TIF)

S4 Fig Nasal immunization with rLdpA-chitin complex induced a Th2 immune response in C57BL/6 mice. (A) Recombinant LdpA (rLdpA) (10 μg), chitin (100 μg), and rLdpA–chitin complex (rLdpA, 10 μg; chitin, 100 μg) were administered intranasally to C57BL/6 mice three times. All animals were sacrificed at 8 h (B) or 72 h (B, C, D, E, and F) after the last nasal administration, and samples were obtained. (B) The numbers of total cells, neutrophils, and eosinophils in bronchoalveolar lavage (BAL) fluid were measured by flow cytometry. (C) KC/CXCL1 and CCL11/Eotaxin-1 protein levels in the lung tissue lysate were measured by enzyme-linked immunosorbent assay (ELISA). (D) IL-4, IL-5, and IFN-γ secretion by spleen cells after *ex vivo* re-stimulation with or without 10 μg/mL rLdpA. (E) Histological examination of lung tissues stained with periodic acid–Schiff (PAS). Scale bar, 100 μm. (F) Levels of serum LdpA-specific IgG1, IgG2a, and IgE measured by indirect ELISA. Data are shown as the mean ± standard deviation (SD) (*n* = 6 mice/group). Each symbol represents an individual sample. *P < 0.05, **P < 0.01, and ***P < 0.001 by one-way ANOVA with *post hoc* Tukey–Kramer test.
(TIF)

S5 Fig IL-1β secretion in BMDCs is not induced by the rLdpA-chitin complex. BMDCs were incubated with rLdpA (0.2–25 μg/mL), chitin particles (2–250 μg/mL), rLdpA-chitin complex (rLdpA, 0.008–1 μg/mL; chitin, 50 μg/mL), or PBS (vehicle control), and IL-1β levels in the culture supernatant were measured by enzyme-linked immunosorbent assay (ELISA).
(TIF)

## References

[1] Vos T, Lim SS, Abbafati C, Abbas KM, Abbasi M, Abbasifard M, et al. Global burden of 369 diseases and injuries in 204 countries and territories, 1990–2019: a systematic analysis for the Global Burden of Disease Study 2019. Lancet. 2020;396(10262):1562. Epub 20201023. doi: 10.1016/S0140-6736(20)30925-9 . 33069326 PMC7567026

[2] Chaudhary N, Marr KA. Impact of *Aspergillus fumigatus* in allergic airway diseases. Clin Transl Allergy. 2011;1(1):4. Epub 20110610. doi: 10.1186/2045-7022-1-4 . 22410255 PMC3294627

[3] Denning DW, O’Driscoll BR, Hogaboam CM, Bowyer P, Niven RM. The link between fungi and severe asthma: a summary of the evidence. Eur Respir J. 2006;27(3):615–26. doi: 10.1183/09031936.06.00074705 . 16507864

[4] O’Driscoll BR, Powell G, Chew F, Niven RM, Miles JF, Vyas A, et al. Comparison of skin prick tests with specific serum immunoglobulin E in the diagnosis of fungal sensitization in patients with severe asthma. Clin Exp Allergy. 2009;39(11):1677–83. Epub 20090818. doi: 10.1111/j.1365-2222.2009.03339.x . 19689458

[5] Agarwal R, Gupta D. Severe asthma and fungi: current evidence. Med Mycol. 2011;49 Suppl 1:S150–7. Epub 20100722. doi: 10.3109/13693786.2010.504752 . 20662637

[6] Katzenstein AL, Sale SR, Greenberger PA. Allergic *Aspergillus* sinusitis: a newly recognized form of sinusitis. J Allergy Clin Immunol. 1983;72(1):89–93. doi: 10.1016/0091-6749(83)90057-x . 6853933

[7] Ghosh S, Hoselton SA, Schuh JM. Allergic Inflammation in *Aspergillus fumigatus*-Induced Fungal Asthma. Curr Allergy Asthma Rep. 2015;15(10):59. doi: 10.1007/s11882-015-0561-x . 26288940

[8] Greenberger PA. Allergic bronchopulmonary aspergillosis. J Allergy Clin Immunol. 2002;110(5):685–92. doi: 10.1067/mai.2002.130179 . 12417875

[9] Knutsen AP. Allergic bronchopulmonary aspergillosis. Clin Exp Allergy. 2015;45(2):298–9. doi: 10.1111/cea.12459 . 25623505

[10] O’Dea EM, Amarsaikhan N, Li H, Downey J, Steele E, Van Dyken SJ, et al. Eosinophils are recruited in response to chitin exposure and enhance Th2-mediated immune pathology in *Aspergillus fumigatus* infection. Infect Immun. 2014;82(8):3199–205. Epub 20140519. doi: 10.1128/IAI.01990-14 . 24842927 PMC4136210

[11] Amarsaikhan N, O’Dea EM, Tsoggerel A, Templeton SP. Lung eosinophil recruitment in response to *Aspergillus fumigatus* is correlated with fungal cell wall composition and requires gammadelta T cells. Microbes Infect. 2017;19(7–8):422–31. Epub 20170525. doi: 10.1016/j.micinf.2017.05.001 . 28552410 PMC5523131

[12] Lee CG, Da Silva CA, Lee JY, Hartl D, Elias JA. Chitin regulation of immune responses: an old molecule with new roles. Curr Opin Immunol. 2008;20(6):684–9. Epub 20081101. doi: 10.1016/j.coi.2008.10.002 . 18938241 PMC2605627

[13] Kogiso M, Nishiyama A, Shinohara T, Nakamura M, Mizoguchi E, Misawa Y, et al. Chitin particles induce size-dependent but carbohydrate-independent innate eosinophilia. J Leukoc Biol. 2011;90(1):167–76. Epub 20110329. doi: 10.1189/jlb.1110624 . 21447645 PMC3114598

[14] Da Silva CA, Chalouni C, Williams A, Hartl D, Lee CG, Elias JA. Chitin is a size-dependent regulator of macrophage TNF and IL-10 production. J Immunol. 2009;182(6):3573–82. doi: 10.4049/jimmunol.0802113 . 19265136

[15] Alvarez FJ. The effect of chitin size, shape, source and purification method on immune recognition. Molecules. 2014;19(4):4433–51. Epub 20140410. doi: 10.3390/molecules19044433 . 24727416 PMC6271096

[16] Nishiyama A, Tsuji S, Yamashita M, Henriksen RA, Myrvik QN, Shibata Y. Phagocytosis of N-acetyl-D-glucosamine particles, a Th1 adjuvant, by RAW 264.7 cells results in MAPK activation and TNF-alpha, but not IL-10, production. Cell Immunol. 2006;239(2):103–12. Epub 20060616. doi: 10.1016/j.cellimm.2006.04.003 . 16781693

[17] Shibata Y, Metzger WJ, Myrvik QN. Chitin particle-induced cell-mediated immunity is inhibited by soluble mannan: mannose receptor-mediated phagocytosis initiates IL-12 production. J Immunol. 1997;159(5):2462–7. . 9278339

[18] Davis S, Cirone AM, Menzie J, Russell F, Dorey CK, Shibata Y, et al. Phagocytosis-mediated M1 activation by chitin but not by chitosan. Am J Physiol Cell Physiol. 2018;315(1):C62–C72. Epub 20180502. doi: 10.1152/ajpcell.00268.2017 . 29719169 PMC6087726

[19] Van Dyken SJ, Mohapatra A, Nussbaum JC, Molofsky AB, Thornton EE, Ziegler SF, et al. Chitin activates parallel immune modules that direct distinct inflammatory responses via innate lymphoid type 2 and gammadelta T cells. Immunity. 2014;40(3):414–24. Epub 20140313. doi: 10.1016/j.immuni.2014.02.003 . 24631157 PMC4019510

[20] Yasuda K, Muto T, Kawagoe T, Matsumoto M, Sasaki Y, Matsushita K, et al. Contribution of IL-33-activated type II innate lymphoid cells to pulmonary eosinophilia in intestinal nematode-infected mice. Proc Natl Acad Sci U S A. 2012;109(9):3451–6. Epub 20120213. doi: 10.1073/pnas.1201042109 . 22331917 PMC3295287

[21] Reese TA, Liang HE, Tager AM, Luster AD, Van Rooijen N, Voehringer D, et al. Chitin induces accumulation in tissue of innate immune cells associated with allergy. Nature. 2007;447(7140):92–6. Epub 20070422. doi: 10.1038/nature05746 . 17450126 PMC2527589

[22] Da Silva CA, Pochard P, Lee CG, Elias JA. Chitin particles are multifaceted immune adjuvants. Am J Respir Crit Care Med. 2010;182(12):1482–91. Epub 20100723. doi: 10.1164/rccm.200912-1877OC . 20656945 PMC3029935

[23] Shibata Y, Honda I, Justice JP, Van Scott MR, Nakamura RM, Myrvik QN. Th1 adjuvant N-acetyl-D-glucosamine polymer up-regulates Th1 immunity but down-regulates Th2 immunity against a mycobacterial protein (MPB-59) in interleukin-10-knockout and wild-type mice. Infect Immun. 2001;69(10):6123–30. doi: 10.1128/IAI.69.10.6123-6130.2001 . 11553551 PMC98742

[24] Hasegawa H, Ichinohe T, Strong P, Watanabe I, Ito S, Tamura S, et al. Protection against influenza virus infection by intranasal administration of hemagglutinin vaccine with chitin microparticles as an adjuvant. J Med Virol. 2005;75(1):130–6. doi: 10.1002/jmv.20247 . 15543590

[25] Hamajima K, Kojima Y, Matsui K, Toda Y, Jounai N, Ozaki T, et al. Chitin Micro-Particles (CMP): a useful adjuvant for inducing viral specific immunity when delivered intranasally with an HIV-DNA vaccine. Viral Immunol. 2003;16(4):541–7. doi: 10.1089/088282403771926355 . 14733740

[26] Baaten BJG Clarke B, Strong P, Hou S. Nasal mucosal administration of chitin microparticles boosts innate immunity against influenza A virus in the local pulmonary tissue. Vaccine. 2010;28(25):4130–7. Epub 20100428. doi: 10.1016/j.vaccine.2010.04.026 . 20433805

[27] Arae K, Morita H, Unno H, Motomura K, Toyama S, Okada N, et al. Chitin promotes antigen-specific Th2 cell-mediated murine asthma through induction of IL-33-mediated IL-1beta production by DCs. Sci Rep. 2018;8(1):11721. Epub 20180806. doi: 10.1038/s41598-018-30259-2 . 30082755 PMC6079063

[28] Saijo S, Ikeda S, Yamabe K, Kakuta S, Ishigame H, Akitsu A, et al. Dectin-2 recognition of alpha-mannans and induction of Th17 cell differentiation is essential for host defense against *Candida albicans*. Immunity. 2010;32(5):681–91. Epub 20100520. doi: 10.1016/j.immuni.2010.05.001 . 20493731

[29] Geijtenbeek TB, Gringhuis SI. C-type lectin receptors in the control of T helper cell differentiation. Nat Rev Immunol. 2016;16(7):433–48. Epub 20160613. doi: 10.1038/nri.2016.55 . 27291962

[30] Barrett NA, Maekawa A, Rahman OM, Austen KF, Kanaoka Y. Dectin-2 recognition of house dust mite triggers cysteinyl leukotriene generation by dendritic cells. J Immunol. 2009;182(2):1119–28. doi: 10.4049/jimmunol.182.2.1119 . 19124755 PMC3682801

[31] Parsons MW, Li L, Wallace AM, Lee MJ, Katz HR, Fernandez JM, et al. Dectin-2 regulates the effector phase of house dust mite-elicited pulmonary inflammation independently from its role in sensitization. J Immunol. 2014;192(4):1361–71. Epub 20140122. doi: 10.4049/jimmunol.1301809 . 24453247 PMC4024442

[32] Barrett NA, Rahman OM, Fernandez JM, Parsons MW, Xing W, Austen KF, et al. Dectin-2 mediates Th2 immunity through the generation of cysteinyl leukotrienes. J Exp Med. 2011;208(3):593–604. Epub 20110228. doi: 10.1084/jem.20100793 . 21357742 PMC3058587

[33] Muraosa Y, Toyotome T, Yahiro M, Kamei K. Characterisation of novel-cell-wall LysM-domain proteins LdpA and LdpB from the human pathogenic fungus *Aspergillus fumigatus*. Sci Rep. 2019;9(1):3345. Epub 20190304. doi: 10.1038/s41598-019-40039-1 . 30833675 PMC6399445

[34] Steentoft C, Vakhrushev SY, Joshi HJ, Kong Y, Vester-Christensen MB, Schjoldager KT, et al. Precision mapping of the human O-GalNAc glycoproteome through SimpleCell technology. EMBO J. 2013;32(10):1478–88. Epub 20130412. doi: 10.1038/emboj.2013.79 . 23584533 PMC3655468

[35] Edge ASB Faltynek CR, Hof L, Reichert LE Jr., Weber P. Deglycosylation of glycoproteins by trifluoromethanesulfonic acid. Anal Biochem. 1981;118(1):131–7. doi: 10.1016/0003-2697(81)90168-8 . 6175244

[36] Edge ASB Deglycosylation of glycoproteins with trifluoromethanesulphonic acid: elucidation of molecular structure and function. Biochem J. 2003;376(Pt 2):339–50. doi: 10.1042/BJ20030673 . 12974674 PMC1223790

[37] Ishikawa T, Itoh F, Yoshida S, Saijo S, Matsuzawa T, Gonoi T, et al. Identification of distinct ligands for the C-type lectin receptors Mincle and Dectin-2 in the pathogenic fungus *Malassezia*. Cell Host Microbe. 2013;13(4):477–88. doi: 10.1016/j.chom.2013.03.008 . 23601109

[38] Di Paolo NC, Shayakhmetov DM. Interleukin 1alpha and the inflammatory process. Nat Immunol. 2016;17(8):906–13. doi: 10.1038/ni.3503 . 27434011 PMC5152572

[39] Gow NAR, Latge JP, Munro CA. The Fungal Cell Wall: Structure, Biosynthesis, and Function. Microbiol Spectr. 2017;5(3). doi: 10.1128/microbiolspec.FUNK-0035-2016 . 28513415 PMC11687499

[40] Ruiz-Herrera J, Elorza MV, Valentin E, Sentandreu R. Molecular organization of the cell wall of *Candida albicans* and its relation to pathogenicity. FEMS Yeast Res. 2006;6(1):14–29. doi: 10.1111/j.1567-1364.2005.00017.x . 16423067

[41] Latge JP. Tasting the fungal cell wall. Cell Microbiol. 2010;12(7):863–72. Epub 20100506. doi: 10.1111/j.1462-5822.2010.01474.x . 20482553

[42] Jin C. Protein Glycosylation in *Aspergillus fumigatus* Is Essential for Cell Wall Synthesis and Serves as a Promising Model of Multicellular Eukaryotic Development. Int J Microbiol. 2012;2012:654251. Epub 20110928. doi: 10.1155/2012/654251 . 21977037 PMC3184424

[43] Goto M. Protein O-glycosylation in fungi: diverse structures and multiple functions. Biosci Biotechnol Biochem. 2007;71(6):1415–27. doi: 10.1271/bbb.70080 . 17587671

[44] Trimble RB, Lubowski C, Hauer CR 3rd, Stack R, McNaughton L, Gemmill TR, et al. Characterization of *N*- and *O*-linked glycosylation of recombinant human bile salt-stimulated lipase secreted by *Pichia pastoris*. Glycobiology. 2004;14(3):265–74. Epub 20031223. doi: 10.1093/glycob/cwh036 . 14693913

[45] Kudoh A, Okawa Y, Shibata N. Significant structural change in both *O*- and *N*-linked carbohydrate moieties of the antigenic galactomannan from *Aspergillus fumigatus* grown under different culture conditions. Glycobiology. 2015;25(1):74–87. Epub 20140903. doi: 10.1093/glycob/cwu091 . 25187160

[46] Reedy JL, Crossen AJ, Negoro PE, Harding HB, Ward RA, Vargas-Blanco DA, et al. The C-Type Lectin Receptor Dectin-2 Is a Receptor for *Aspergillus fumigatus* Galactomannan. mBio. 2023;14(1):e0318422. Epub 20230104. doi: 10.1128/mbio.03184-22 . 36598192 PMC9973300

[47] Dam TK, Brewer CF. Lectins as pattern recognition molecules: the effects of epitope density in innate immunity. Glycobiology. 2010;20(3):270–9. doi: 10.1093/glycob/cwp186 19939826

[48] Dam TK, Brewer CF. Maintenance of cell surface glycan density by lectin-glycan interactions: a homeostatic and innate immune regulatory mechanism. Glycobiology. 2010;20(9):1061–4. doi: 10.1093/glycob/cwq084 20548106

[49] Norimoto A, Hirose K, Iwata A, Tamachi T, Yokota M, Takahashi K, et al. Dectin-2 promotes house dust mite-induced T helper type 2 and type 17 cell differentiation and allergic airway inflammation in mice. Am J Respir Cell Mol Biol. 2014;51(2):201–9. doi: 10.1165/rcmb.2013-0522OC . 24588637

[50] Mora-Montes HM, Netea MG, Ferwerda G, Lenardon MD, Brown GD, Mistry AR, et al. Recognition and blocking of innate immunity cells by *Candida albicans* chitin. Infect Immun. 2011;79(5):1961–70. Epub 20110228. doi: 10.1128/IAI.01282-10 . 21357722 PMC3088140

[51] Mantovani A, Dinarello CA, Molgora M, Garlanda C. Interleukin-1 and Related Cytokines in the Regulation of Inflammation and Immunity. Immunity. 2019;50(4):778–95. doi: 10.1016/j.immuni.2019.03.012 . 30995499 PMC7174020

[52] Kuroda E, Ozasa K, Temizoz B, Ohata K, Koo CX, Kanuma T, et al. Inhaled Fine Particles Induce Alveolar Macrophage Death and Interleukin-1alpha Release to Promote Inducible Bronchus-Associated Lymphoid Tissue Formation. Immunity. 2016;45(6):1299–310. doi: 10.1016/j.immuni.2016.11.010 . 28002730

[53] Rabolli V, Badissi AA, Devosse R, Uwambayinema F, Yakoub Y, Palmai-Pallag M, et al. The alarmin IL-1alpha is a master cytokine in acute lung inflammation induced by silica micro- and nanoparticles. Part Fibre Toxicol. 2014;11(1):69. Epub 20141213. doi: 10.1186/s12989-014-0069-x . 25497724 PMC4279463

[54] Dinarello CA. Immunological and inflammatory functions of the interleukin-1 family. Annu Rev Immunol. 2009;27:519–50. doi: 10.1146/annurev.immunol.021908.132612 . 19302047

[55] Takeshige T, Harada N, Harada S, Ishimori A, Katsura Y, Sasano H, et al. Chitin induces steroid-resistant airway inflammation and airway hyperresponsiveness in mice. Allergol Int. 2021;70(3):343–50. Epub 20210225. doi: 10.1016/j.alit.2020.12.004 . 33640239

[56] Munoz-Wolf N, Lavelle EC. A Guide to IL-1 family cytokines in adjuvanticity. FEBS J. 2018;285(13):2377–401. Epub 20180503. doi: 10.1111/febs.14467 . 29656546

[57] Bueter CL, Lee CK, Rathinam VAK, Healy GJ, Taron CH, Specht CA, et al. Chitosan but not chitin activates the inflammasome by a mechanism dependent upon phagocytosis. J Biol Chem. 2011;286(41):35447–55. Epub 20110823. doi: 10.1074/jbc.M111.274936 . 21862582 PMC3195641

[58] Da Silva CA, Hartl D, Liu W, Lee CG, Elias JA. TLR-2 and IL-17A in chitin-induced macrophage activation and acute inflammation. J Immunol. 2008;181(6):4279–86. doi: 10.4049/jimmunol.181.6.4279 . 18768886 PMC2577310

[59] Fuchs K, Cardona Gloria Y, Wolz OO, Herster F, Sharma L, Dillen CA, et al. The fungal ligand chitin directly binds TLR2 and triggers inflammation dependent on oligomer size. EMBO Rep. 2018;19(12). Epub 20181018. doi: 10.15252/embr.201846065 . 30337494 PMC6280652

[60] Saijo S, Fujikado N, Furuta T, Chung SH, Kotaki H, Seki K, et al. Dectin-1 is required for host defense against Pneumocystis carinii but not against Candida albicans. Nat Immunol. 2007;8(1):39–46. Epub 20061210. doi: 10.1038/ni1425 . 17159982

[61] Miyake Y, Toyonaga K, Mori D, Kakuta S, Hoshino Y, Oyamada A, et al. C-type lectin MCL is an FcRgamma-coupled receptor that mediates the adjuvanticity of mycobacterial cord factor. Immunity. 2013;38(5):1050–62. Epub 20130418. doi: 10.1016/j.immuni.2013.03.010 . 23602766

[62] Yamasaki S, Matsumoto M, Takeuchi O, Matsuzawa T, Ishikawa E, Sakuma M, et al. C-type lectin Mincle is an activating receptor for pathogenic fungus, *Malassezia*. Proc Natl Acad Sci U S A. 2009;106(6):1897–902. Epub 20090126. doi: 10.1073/pnas.0805177106 . 19171887 PMC2644135

[63] Adachi O, Kawai T, Takeda K, Matsumoto M, Tsutsui H, Sakagami M, et al. Targeted disruption of the MyD88 gene results in loss of IL-1- and IL-18-mediated function. Immunity. 1998;9(1):143–50. doi: 10.1016/s1074-7613(00)80596-8 . 9697844

[64] Hara H, Ishihara C, Takeuchi A, Imanishi T, Xue L, Morris SW, et al. The adaptor protein CARD9 is essential for the activation of myeloid cells through ITAM-associated and Toll-like receptors. Nat Immunol. 2007;8(6):619–29. Epub 20070507. doi: 10.1038/ni1466 . 17486093

[65] Yamasaki S, Ishikawa E, Sakuma M, Hara H, Ogata K, Saito T. Mincle is an ITAM-coupled activating receptor that senses damaged cells. Nat Immunol. 2008;9(10):1179–88. Epub 20080907. doi: 10.1038/ni.1651 . 18776906

[66] Roy RM, Wuthrich M, Klein BS. Chitin elicits CCL2 from airway epithelial cells and induces CCR2-dependent innate allergic inflammation in the lung. J Immunol. 2012;189(5):2545–52. Epub 20120730. doi: 10.4049/jimmunol.1200689 . 22851704 PMC3424300

[67] Lutz MB, Kukutsch N, Ogilvie AL, Rossner S, Koch F, Romani N, et al. An advanced culture method for generating large quantities of highly pure dendritic cells from mouse bone marrow. J Immunol Methods. 1999;223(1):77–92. doi: 10.1016/s0022-1759(98)00204-x 10037236

