# Supplementary material for: Fungal chitin-binding glycoprotein induces Dectin-2-mediated allergic airway inflammation synergistically with chitin
Source: PLoS Pathog. 2024 Jan 3;20(1):e1011878. doi: 10.1371/journal.ppat.1011878 (PMC10763971; doi:10.1371/journal.ppat.1011878)
Supplement: S1 Table — (PDF) [file ppat.1011878.s001.pdf]

Supplementary Table S1. Demographic Characteristics of the Participants and Healthy controls

**Groups**	**No. of patients**	**Age (Mean ± SD)**	**Sex（Male/Female)**
IPA	11	50.1 ± 11.7	5/6
CPA	10	65.1 ± 9.5	3/7
ABPA	5	58.4 ± 12.0	1/4
Control	10	44.6 ± 14.2	6/4

ABPA, allergic bronchopulmonary aspergillosis; CPA, chronic pulmonary aspergillosis; IPA, invasive pulmonary aspergillosis.
