# Supplementary material for: Fungal chitin-binding glycoprotein induces Dectin-2-mediated allergic airway inflammation synergistically with chitin
Source: PLoS Pathog. 2024 Jan 3;20(1):e1011878. doi: 10.1371/journal.ppat.1011878 (PMC10763971; doi:10.1371/journal.ppat.1011878)
Supplement: S2 Table — (PDF) [file ppat.1011878.s002.pdf]

Supplementary Table S2. List of *Pichia pastoris* Strains Used in This Study

**Strains**	**Description used in this study**	**Relevant characteristics**	**Source**
GS115	GS115	*his4*	Thermo Fisher Scientific
YMPP 0203	GS115-LdpA	P_{aox1}-*αfactor-ldpA-c-Myc-6xHis*-T_{aox1}, *ZeoR*, *his4*	Present study
