# Supplementary material for: Fungal chitin-binding glycoprotein induces Dectin-2-mediated allergic airway inflammation synergistically with chitin
Source: PLoS Pathog. 2024 Jan 3;20(1):e1011878. doi: 10.1371/journal.ppat.1011878 (PMC10763971; doi:10.1371/journal.ppat.1011878)
Supplement: S3 Table — (PDF) [file ppat.1011878.s003.pdf]

Supplementary Table S3. Plasmids Used in This Study

**Plasmid**	**Relevant characteristics**	**Source**
pCR2.1-*ldpA*	*ldpA* CDS cloned into pCR2.1-TOPO, Km^R	Muraosa *et al.*, 2019 [1]
pPICZαC	*Pichia pastoris* homologous recombination vector, Zeo^R, P_{aox1}-*α-factor-c-Myc-6xhis*-T_{aox1}	Thermo Fisher Scientific
pPICZαC-*ldpA*	*ldpA* cloned into pPICZαC, Zeo^R, P_{aox1}-*α-factor-ldpA-c-Myc-6xhis*-T_{aox1}	Present study

References

1. Muraosa Y, Toyotome T, Yahiro M, Kamei K. Characterisation of novel-cell-wall LysM-domain proteins LdpA and LdpB from the human pathogenic fungus Aspergillus fumigatus. Sci Rep. 2019;9(1):3345. Epub 20190304. doi: 10.1038/s41598-019-40039-1. PubMed PMID: 30833675; PubMed Central PMCID: PMC6399445.
