# Supplementary material for: Fungal chitin-binding glycoprotein induces Dectin-2-mediated allergic airway inflammation synergistically with chitin
Source: PLoS Pathog. 2024 Jan 3;20(1):e1011878. doi: 10.1371/journal.ppat.1011878 (PMC10763971; doi:10.1371/journal.ppat.1011878)
Supplement: S4 Table — (PDF) [file ppat.1011878.s004.pdf]

Supplementary Table S4. Primers Used for Quantitative RT-PCR in This Study

**Species**	**Gene**	**Primers (5′ → 3′)**	**Primers (5′ → 3′)**
Mouse	*Tnf-α*	CTTCTGTCTACTGAACTTCGGG	CAGGCTTGTCACTCGAATTTTG
Mouse	*Il-1α*	GTCGGCAAAGAAATCAAGATGG	AGTGAGCCATAGCTTGCATC
Mouse	*Il-1β*	TCCTGTGTAATGAAAGACGGC	ACTCCACTTTGCTCTTGACTTC
Mouse	*Il-12 p35*	GGTCCAGCATGTGTCAATCA	ATGTCATCTGTGGTCTTCAGC
Mouse	*Il-12 p40*	ACTCCCCATTCCTACTTCTCC	CATTCCCGCCTTTGCATTG
Mouse	*Il-6*	CAAAGCCAGAGTCCTTCAGAG	GTCCTTAGCCACTCCTTCTG
Mouse	*Kc/Cxcl1*	TGGCTGGGATTCACCTCAAG	CAGACAGGTGCCATCAGAGC
Mouse	*Mip-2/Cxcl2*	AGACAGAAGTCATAGCCACTCTCAAG	CCTCCTTTCCAGGTCAGTTAGC
Mouse	*Ccl11/Eotaxin-1*	TCCACAGCGCTTCTATTCCT	CTATGGCTTTCAGGGTGCAT
Mouse	*Muc5b*	CTGGCACCTGCTCTGTGCA	CACTGCTTTGAGGCAGTTCT
Mouse	*Chia1/AMCase*	CTGCTGGGCCCTATACCAG	CCTCAGTGGCTCCACTTCTC
Mouse	*Gapdh*	CTCCCACTCTTCCACCTTCG	CCACCACCCTGTTGCTGTAG
