# Supplementary figures and images for: Fungal chitin-binding glycoprotein induces Dectin-2-mediated allergic airway inflammation synergistically with chitin
Source: PLoS Pathog. 2024 Jan 3;20(1):e1011878. doi: 10.1371/journal.ppat.1011878 (PMC10763971; doi:10.1371/journal.ppat.1011878)

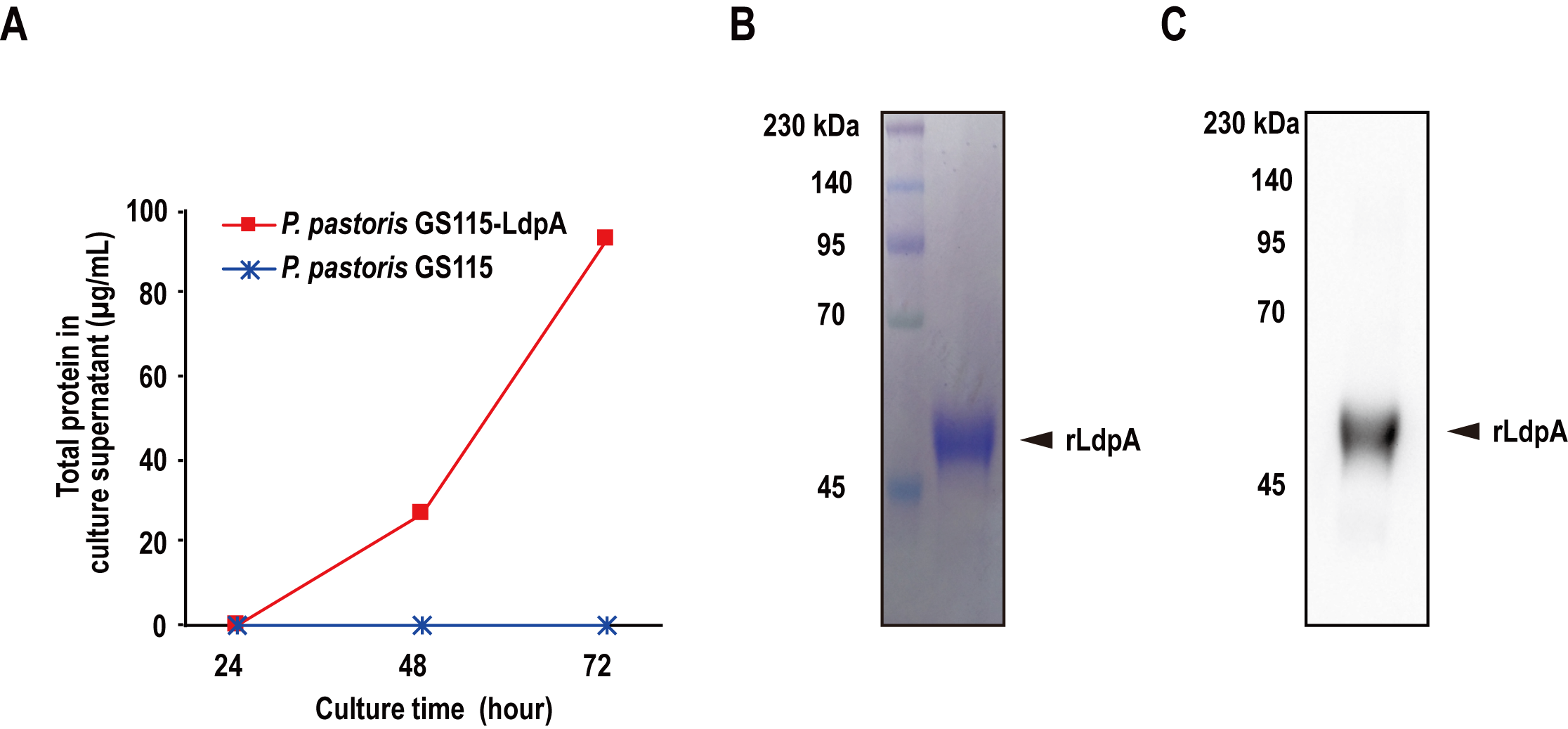

Supplement: S1 Fig — (A) Total protein levels in the culture supernatant. P. pastoris GS115 and ldpA transformant GS115-LdpA were cultured in buffered methanol medium (BMM), and protein expression was induced by the addition of methanol. Total protein levels in the culture supernatant were measured by BCA protein assay. (B, C) Purified recombinant LdpA (rLdpA) was confirmed by SDS-PAGE (B) and Western blotting analysis using anti-c-Myc antibody and HRP-conjugated secondary antibody (C). (TIF) [file ppat.1011878.s005.tif]

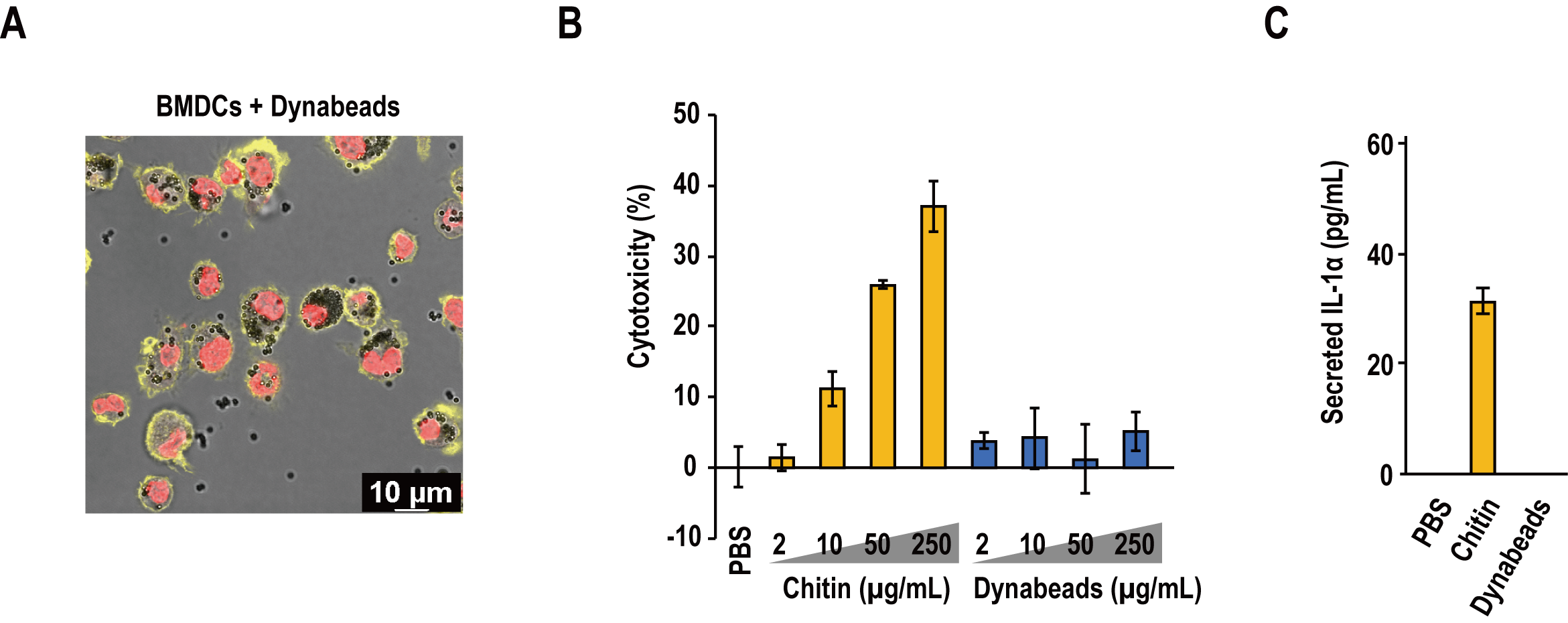

Supplement: S2 Fig — BMDCs from C57BL/6 mice were incubated with Dynabeads, chitin, or PBS for 24 h. (A) After washing with PBS, cells were stained with Alexa Fluor 555 Phalloidin (yellow), nuclei were stained with NucRed Live 647 ReadyProbes Reagent (red), and observed by confocal laser scanning microscopy. (B) Cytotoxicity was assessed by measuring the release of LDH. (C) IL-1α protein levels in the culture supernatant were measured by ELISA. (TIF) [file ppat.1011878.s006.tif]

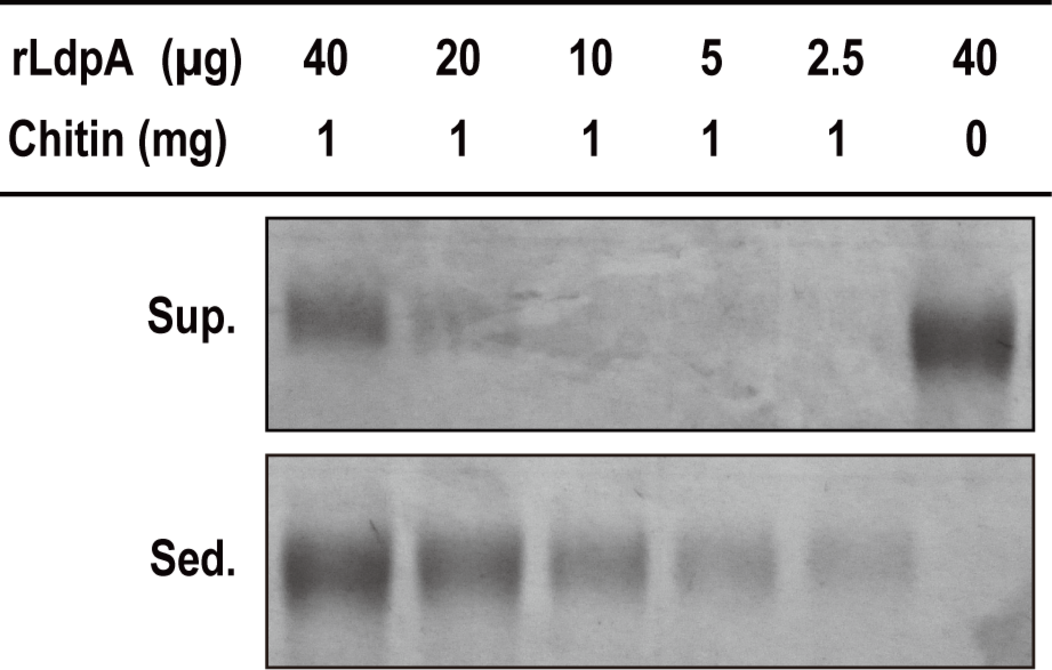

Supplement: S3 Fig — After incubation of serially diluted rLdpA (2.5–40 μg) with chitin (1 mg) in 100 μL of PBS, rLdpA in the supernatant (sup.) and sediment (sed.) was evaluated by SDS-PAGE. (TIF) [file ppat.1011878.s007.tif]

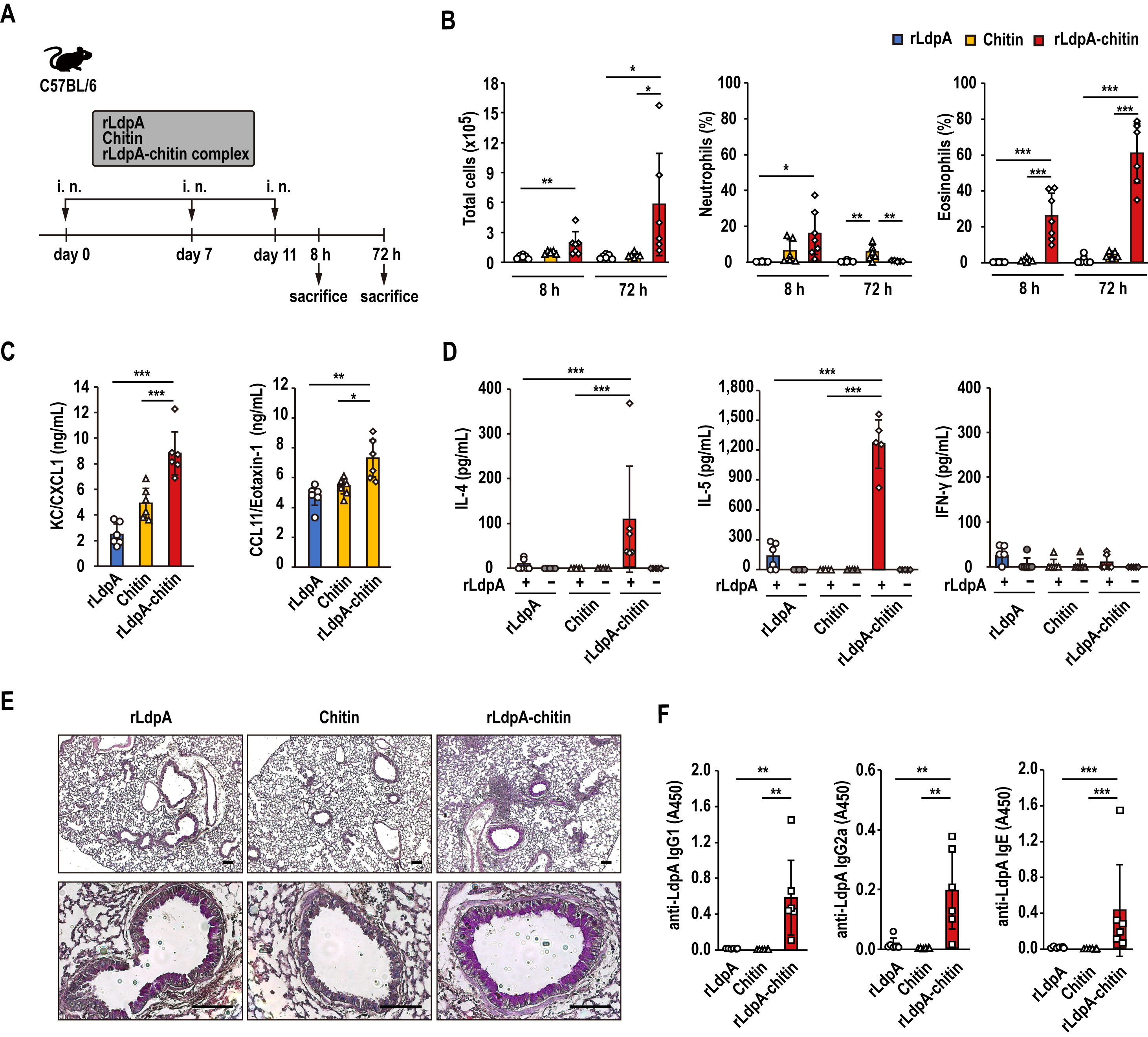

Supplement: S4 Fig — (A) Recombinant LdpA (rLdpA) (10 μg), chitin (100 μg), and rLdpA–chitin complex (rLdpA, 10 μg; chitin, 100 μg) were administered intranasally to C57BL/6 mice three times. All animals were sacrificed at 8 h (B) or 72 h (B, C, D, E, and F) after the last nasal administration, and samples were obtained. (B) The numbers of total cells, neutrophils, and eosinophils in bronchoalveolar lavage (BAL) fluid were measured by flow cytometry. (C) KC/CXCL1 and CCL11/Eotaxin-1 protein levels in the lung tissue lysate were measured by enzyme-linked immunosorbent assay (ELISA). (D) IL-4, IL-5, and IFN-γ secretion by spleen cells after ex vivo re-stimulation with or without 10 μg/mL rLdpA. (E) Histological examination of lung tissues stained with periodic acid–Schiff (PAS). Scale bar, 100 μm. (F) Levels of serum LdpA-specific IgG1, IgG2a, and IgE measured by indirect ELISA. Data are shown as the mean ± standard deviation (SD) (n = 6 mice/group). Each symbol represents an individual sample. *P < 0.05, **P < 0.01, and ***P < 0.001 by one-way ANOVA with post hoc Tukey–Kramer test. (TIF) [file ppat.1011878.s008.tif]

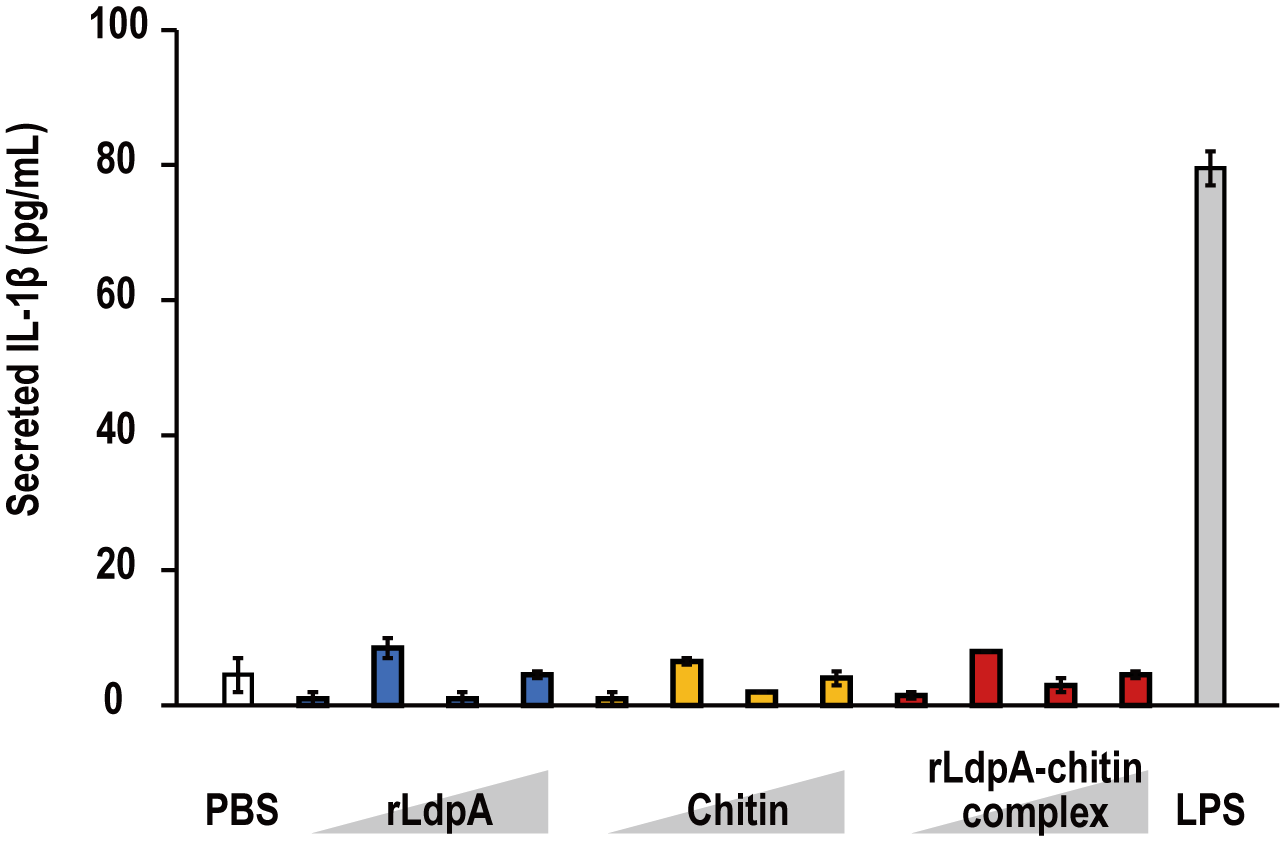

Supplement: S5 Fig — BMDCs were incubated with rLdpA (0.2–25 μg/mL), chitin particles (2–250 μg/mL), rLdpA-chitin complex (rLdpA, 0.008–1 μg/mL; chitin, 50 μg/mL), or PBS (vehicle control), and IL-1β levels in the culture supernatant were measured by enzyme-linked immunosorbent assay (ELISA). (TIF) [file ppat.1011878.s009.tif]
